# Mechanically Recycled Textiles: A Source of Microplastic Fiber Emissions

**DOI:** 10.1021/acs.est.5c14973

**Published:** 2026-01-07

**Authors:** Maria Persson, Juliana Aristéia de Lima, Nawar Kadi, Nils-Krister Persson

**Affiliations:** 1 The Swedish School of Textiles, Faculty of Textiles, Engineering and Business, University of Borås, Borås 501 90, Sweden; 2 Swedish Centre of Resource Recovery, Faculty of Textiles, Engineering and Business, 1802 University of Borås, Borås 501 90, Sweden; 3 Department of Polymer, Fiber and Composite, RISE Research Institutes of Sweden, Borås 504 62, Sweden; 4 Polymer E-textiles, The Swedish School of Textiles, Smart Textiles, Science Park Borås, University of Borås, Borås 501 90, Sweden

**Keywords:** microplastic, microplastic fiber, fiber fragmentation, mechanical recycling, wear simulation, textile durability, dry shedding

## Abstract

Our research found that the shedding of microplastic fibers (MPFs) from textiles is exacerbated by repeated mechanical recycling, raising environmental concerns as the use of recycled fibers increases in industry. This study examined MPF release from fabrics containing 30% mechanically recycled polyester fibers subjected to one, two, or three recycling cycles, compared to primary (virgin) polyester (PES). Shedding was assessed under both simulated wear and laundering conditions using Martindale, ICI Pilling Box, and ISO 4484–1:2023 (microplastic from textile sources) protocols. Laundering tests showed no clear difference in MPF release between primary PES and once-recycled PES (rPES-1; ∼ 1.4-fold). In contrast, fabrics with fibers recycled twice (rPES-2) and three times (rPES-3) released about 4.3-fold and 6.2-fold more MPFs than PES, respectively. Fiber release was different under dry-state abrasion than in laundry tests, highlighting the limitations of current wet-state focused assessments. Progressive fiber fragmentation and increased yarn hairiness suggest cumulative structural degradation with each recycling cycle. These findings underscore the need for standardized dry-state shedding assessments and improved recycling strategies to mitigate MPF emissions. While mechanical recycling remains environmentally preferable to uncontrolled disposal, these findings reveal a trade-off in the form of increased MPF release after multiple recycling cycles, which could be mitigated through improved recycling processes and fabric design. Achieving a balance between textile circularity and environmental sustainability remains a critical challenge for the industry.

## Graphical abstract

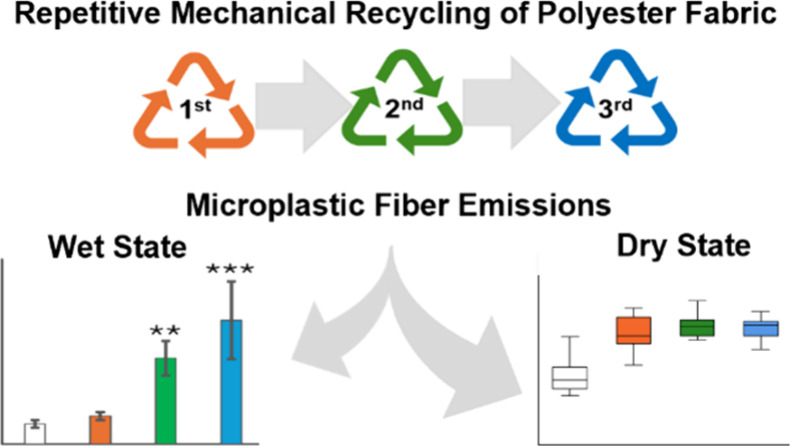

## Introduction

Microplastic fibers (MPFs), described as fibrous or thread-like plastic fragments up to 15 mm in length and with a length-to-diameter ratio greater than 3 have emerged as a critical environmental concern due to their widespread distribution and persistence across aquatic, terrestrial, and atmospheric ecosystems.^1^ Synthetic textiles, particularly those made from polyester and polyamide, estimated to contribute up to 35% of primary microplastics in marine environment, amounting to approximately 0.5 million tons annually^2^ with laundering alone capable of releasing hundreds of thousands of microfibers per wash.^3–6^ Textile fiber shedding is particularly pronounced during the first few laundering cycles of new garments.^7^ Once released, MPFs accumulate in the ecosystems, where they act as carriers for toxic pollutants, enter food chains, and raising both ecological and human health concerns.^8^ In terrestrial ecosystems, MPFs disrupt microbial activity and soil chemistry,^9^ while airborne MPF contribute to both indoor and outdoor pollution and pose inhalation risks to humans.^10^ As the problem of microplastics pollution escalates, the European Commission has introduced regulatory measures to address microplastic contamination more comprehensively. For example, as of 17 October 2023, a ban was implemented on microplastics intentionally added to products such as glitter.^2^

Simultaneously, regulatory frameworks targeting the textile sector have been developed to embed circular economy principles into production and waste management systems. These initiatives aim to reduce textile waste, favor reuse, improve recyclability, and minimize MPF emissions through sustainable design and end-of-life strategies.^11^ To support these goals, textile recycling typically follows one of the three primary routes: (1) mechanical recycling, (2) thermo-mechanical recycling, or (3) chemical recycling, or a combination of these processes. However, each method has distinct implications for fiber quality, energy use and environmental impact.^12,13^ In practice, as of today, the most widely adopted approach for managing textile waste is mechanical recycling, which involves the physical breakdown of postconsumer fabrics into reusable fibers.^14^ Although not explicitly favored in the regulatory framework over other recycling methods, mechanical recycling is frequently applied due to its accessibility, cost-effectiveness, and compatibility with existing industrial infrastructure. However, this method is not without limitations. As the name implies, mechanical recycling involves the use of large forces that tear apart and damage textile fibers, resulting in shorter fiber length and reduced fiber cohesion, which may compromise the quality of the recycled material.^15^ Additionally, the process generates significant amounts of textile dust, which pose health risks and environmental challenges if not properly managed.^16–18^

While MPF mitigation and recyclability have been studied independently, little research has explored how to optimize textile design in way that addresses both challenges simultaneously. Recent innovations, for instance, encompass the use of fiber lubricants to reduce mechanical damage, precision shredding techniques to preserve fiber length and modified spinning processes that enhance the durability of recycled yarns.^15^ Nonetheless, inherent trade-offs remain; while tightly woven fabrics can minimize MPF shedding, they often require greater mechanical force during recycling; in contrast, loosely knitted structures, which facilitate easier recycling, tend to release a higher volume of fibers.^19,20^ The mechanical recycling process, along with key stages where MPF release occurs, such as fiber breakdown, yarn formation, fabric production, wear, and laundering, is illustrated in Figure 1. This overview highlights the multiple points at which fiber damage and shedding can be introduced or exacerbated, underscoring the complexity of addressing both recyclability and environmental performance in textile design.

**Figure 1:**
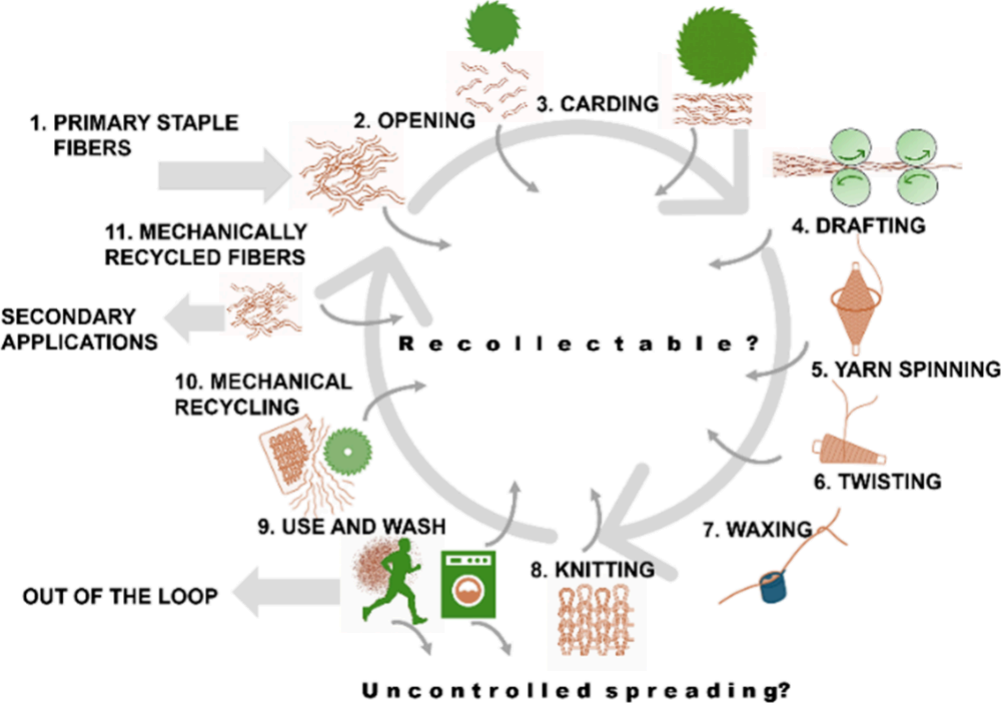
Process flow diagram of mechanical textile recycling, highlighting key stages where microplastic fiber (MPF) release can occur. The illustration outlines both the product life cycle and fiber recovery pathway, including secondary applications and potential out-of-the-loop flows. It also illustrates potential losses (as MPF), divided into a potentially recollectable part since these steps occur within controlled industrial processes and potentially uncontrolled spreading. The figure underscores the importance of addressing MPF release during yarn formation, fabric production, wear, laundering, and secondary use pathways.

Expanding on this issue, the environmental performance of mechanically recycled textiles remains an open question, particularly regarding their potential for MPF shedding. Some studies suggest that recycled yarns with lower tensile strength shed more MPFs during use,^21^ while others report no significant difference between primary (also known as virgin fiber) and recycled fibers. In some cases, recycled fibers have been shown to release longer MPFs, which further fragment into secondary microplastics.^21^ Notably, much of this research has focused on textiles produced from thermo-mechanically recycled PET bottles, which do not accurately reflect the behavior of fiber-to-fiber mechanically recycled textiles.^22^ For example, Özkan and Güdoğdu^23^ reported that knitted fabrics made from recycled PET released 2.3 times more MPFs than those made from primary polyester. Comparable results were reported by Akyildiz et al.,^24^ who observed that rPET fabrics shed more MPFs during laundering compared with primary PET fabrics; however, the study did not specify yarn type (multifilament or spun) or the recycling method used. By contrast, Chandra Manivannan et al.^25^ provided detailed analysis of chemical recycling processes, noting that, despite their potential to divert blended textiles from landfill, these processes can still contribute to microplastic pollution. Together, these findings suggest that differences in material origin or recycling method alone cannot fully explain MPF release patterns, pointing to the influence of other structural or processing factors.

One such factor is yarn hairiness, characterized by fibers protruding from the yarn surface, which has been identified as a critical influence on microplastic fiber emissions.^26^ Increased hairiness generally corresponds to higher fiber shedding due to weaker fiber cohesion and a higher likelihood of surface abrasion and detachment during textile usage and laundering. Several studies have documented the correlation between elevated yarn hairiness and enhanced microplastic release, emphasizing the necessity for controlled yarn manufacturing processes.^26,27^ Mechanical recycling often produces shorter fibers, which contribute to increased yarn hairiness. Despite this, some studies comparing virgin and mechanically recycled PET have not considered yarn hairiness as a variable. For example, Gao et al.^28^ found no differences in MPF release between primary and mechanically recycled PET; however, they did not characterize yarn hairiness or assess the potential influence of incorporating fibers from multiple recycling cycles. This underscores the need to examine not only the material source but also the effects of recycling processes on fiber and yarn characteristics

Building on these insights, it becomes clear that while sustainable textile production is gaining momentum, the specific impact of repeated mechanical recycling on MPF shedding remains largely unexplored. Understanding this relationship is essential for designing recycling systems that support circular economy goals by maximizing material reuse, minimizing environmental harm, and maintaining textile performance. Addressing this knowledge gap, the present study investigates how multiple cycles of mechanical recycling influence MPF shedding under simulated wear and laundering conditions. Standardized abrasion tests (Martindale and ICI Pilling Box) and controlled laundering experiments, following the guidelines of the SS-EN-ISO 4484–1:2023 protocol, were used to quantify MPF release. The findings aim to inform strategies for optimizing mechanical recycling processes that balance environmental performance with recyclability in textile design.

## Materials and Methods

### Reference Fabric Production

To establish a baseline for comparison, a reference fabric was produced using 100% primary polyester (PES) staple fibers. These fibers, measuring nominally 38 mm in length with a linear density of 1.7 dtex, were sourced from Wagenfelder Spinnereien GmbH, Wagenfeld, Germany. A total of 20 g of PES fibers were processed into a sliver, which was subjected to an opening process followed by two rounds of carding, drafting, and ring spinning, as illustrated in Supporting Information Figure S1. The opening was performed using an edge opener from LaRoche (Andritz Laroche, France), while carding, drafting, and ring spinning were conducted using Mesdan machines (337A, 3371, and 310A, respectively) (Mesdan Spa, Italy).

During carding, the fiber web was folded and rotated 90° between rounds to aim for uniform mixing. The drafted web was processed with a draft ratio of 3.57. Four slivers were combined and drafted again, with the weight recorded to monitor fiber loss. The sliver was then ring spun under controlled conditions: a predraft of 2.3, a total draft of 18, and a twist of 581 in the *z*-direction, at a production speed of approximately 10 m per minute. These parameters were standardized for consistency in subsequent recycling iterations. Following ring spinning, two single yarns were twisted together to form a 2-ply yarn using an AG TEK direct Twist-C6 machine (Istanbul, Turkey), applying a twist in the S-direction of 180 twists per meter at a speed of 16.6 m per minute (Figure S1).

The 2-ply yarn was knitted into fabric using a STOLL ADF 530 K 7.2 multigauge machine equipped with a 14-gauge needle bed and a 12-gauge needle (Stoll, Karl Mayer Group, Germany). The fabric structure used was half Milano, a common structure in the textile industry. Prior to knitting, the yarn was waxed with paraffin.

All knitted fabrics were prewashed using a Wascator FOM71 laboratory washing machine (Electrolux AB, Sweden) in accordance with ISO 6330:2021. The washing program 4N was employed at 40 °C to remove spin oils, wax, foreign fiber matter, and other contaminants introduced during the preparation process. Each wash contained 2 kg of polyester fabric and 20 ± 0.5 g of phosphorus-free, type A powder reference detergent (Non-Phosphate Reference Detergent A, James Heal, England). Following washing, the fabrics were flat-dried in a drying cabinet at 50 °C for 2 h.

### Mechanical Recycling and Iterative Processing

Following the production of the reference fabric (PES), the material was subjected to mechanical recycling. The fabric was collected and processed using an edge trim opener by Qingdao Kingtech Machinery Co., Ltd., Qingdao, China, as detailed in a previous publication.^29^ The fabric’s thread orientation was randomized before being fed into the shredder. The shredded fibers were subsequently collected and opened once with the edge opener. This first recycling iteration is referred to as rPES-1.

For each recycling iteration, 30% of the recycled PES fibers were blended with 70% primary (unrecycled) PES fibers before undergoing the same fiber processing steps as the reference fabric (carding, drafting, spinning, knitting, and prewashing). This process was repeated for the second (rPES-2) and third (r-PES-3) cycles, where the material from the second cycle was recycled and blended again with primary PES fibers for the third cycle. The same processing parameters were maintained to ensure comparability across all recycling iterations. For the yarn containing rPES from the third recycling (rPES-3), the humidity increased from approximately 45% to 60% to enable handling of the fibers during the process.

### Fiber Characterization

Fiber length distribution was analyzed for both primary PES fibers after opening and for recycled fibers after shredding and opening, using a fiber length measurement device from TexTechno, Mönchengladbach, Germany. The Fiber Bundle Test method, implemented in the supplier-provided software, was used to determine parameters such as mean length, short fiber count (SFC), and uniformity index. Ten measurements were conducted per sample.

### Yarn Characterization

Yarn tenacity and elongation at break were measured using a Mesdan (S.p.A, Puegnago sul Garda,) 2512A tensile tester under controlled conditions (21 °C and 65% RH). The tensile test utilized pneumatic yarn clamp equipment with a 0.1 kN load cell, a crosshead speed of 500 mm/min, and an initial grip separation of 50 mm, with a pretension of 26 cN. Linear density was determined by weighing 100 m of yarn. Prior to testing, the yarn was conditioned in a climate chamber at 23 °C and 50% relative humidity for at least 40 h. For each sample, 10 individual tensile tests were conducted, and the mean and standard deviation were reported. Yarn hairiness, defined as the degree to which individual fibers protrude from the yarn surface, was assessed using a Mesdan Evenness Tester (Mesdan S.p.A, Puegnago sul Garda, Italy). Measurements were performed on yarn lengths of 20 m, utilizing a take-up roller speed of 8 m/min under standardized laboratory conditions (21 °C and 65% relative humidity). The hairiness index (H) was reported as the cumulative length of fiber protrusions per unit length of yarn. To ensure accuracy and consistency, each yarn type was analyzed in triplicate (n = 3), with mean values and standard deviations reported to reflect surface uniformity and the potential propensity for fiber shedding.

### Fiber Fragment Shedding in a Dry State

Fiber fragment shedding in dry state was evaluated using the ICI pilling box test and the Martindale test.

#### ICI Pilling Box Test

Wear simulation was performed using an ICI pilling box apparatus (James H. Heal & Co. Ltd., Halifax, England) in accordance with SS-EN ISO 12945–1:2020. The apparatus simulates real-life wear conditions by rubbing specimens against one another and a cork-lined interior. Four specimens (125 mm × 125 mm) were cut, folded, and sewn using a continuous polyester filament thread before inversion and mounting onto polyurethane sample tubes. Carbon tape (PELCO Tabs, 12 mm diameter) was affixed diagonally to the pilling box walls. The test was conducted under controlled conditions (20 °C ± 2 °C, 60 ± 10% RH), with samples subjected to 14,400 rotations.

#### Modified Martindale Test

A 6-station Martindale tester (James H. Heal & Co. Ltd., Halifax, England) was used following SS-EN ISO 12945–2:2000, with modifications to prevent contamination from external fibers. Identical fabrics were employed as both specimens and abradants to ensure consistency. Leather sheets (1.5 mm thickness) were used as base underlays instead of standard materials to prevent contamination and material mixing during the analysis. To ensure controlled testing conditions and prevent any unintended external influence, the test was conducted in a custom-made Plexiglas enclosure. The test was conducted under a pressure of 155 g at 125, 500, 1000, 2000, 5000, and 7000 cycles which is according to the ISO 12945–2000 standard. Carbon tape was systematically applied to collect shed fibers, with triplicate testing (n = 9) performed for each fabric type and cycle run.

### Microscopic Analysis of Shed Fiber Fragments

Carbon tape used to collect shed fibers during the ICI Pilling Box and Martindale tests was analyzed under a stereomicroscope to assess fiber fragmentation. Microplastic fiber analysis was performed using a Nikon SMZ800 optical microscope (Nikon Corp, Tokyo, Japan) equipped with NIS-Element image analysis software. Microscopic images were taken at a magnification of × 2. ImageJ (version 1.54) was employed for binary image conversion and area fraction calculations. In this context, area fraction refers to the proportion of the total image area covered by visible microplastic fibers, expressed as a percentage of the total field of view. (See Supporting Information Figure S2 for illustration). The surface of the fibers was characterized by scanning electron microscopy (SEM) using a Zeiss Supra 40 VP SEM with backscattered electrons (BSEs) detector at an acceleration rate of 20 kV in low vacuum.

### Fiber Fragment Shedding During Laundry

Fiber loss was measured following SS-EN ISO 4484–1:2023. Four specimens underwent washing at 40 °C using 360 mL of water for 45 min, without detergent. Filtration was performed using a Millipore fibrous disc filter (Merck Millipore,US CAT No: APFA04700, 1.6 μm pore size, 47 mm diameter). An analytical balance with 0.1 mg precision was used for weight measurements. The percentage of fiber fragment release was calculated using the equation:

$$P_{f}=100\frac{M_{f}}{S_{m1}}$$ 1

Where:

*P*_f_ = percent of fiber released (%)

*M*_f_ = mass of fiber released (g)

*S*_m1_ = mass in grams of specimen before testing (g)

### Statistics

Statistical analyses were conducted using Minitab Statistical Software Version 21.1.1 to assess differences in the area fraction of MPF released from textile samples. Data normality was verified using the Anderson-Darling test; however, the power of this test is limited given the small number of replicates. A one-way ANOVA followed by Tukey’s HSD test was used to compare the MPF release between groups. Statistical significance was defined as α=0.05.

## Results and Discussion

### Mechanically Recycled Fabrics

This study investigated how repeated mechanical recycling affects the MPF shedding potential of knitted PES fabrics under simulated wear and laundering conditions. As textile recycling has gained increasing importance in the textile industry, driven by growing sustainability concerns and regulatory pressure from the European Commission.^11^ Mechanical recycling has emerged as a widely used, though technically limited in terms of quality, approach for achieving fiber-to-fiber circularity. One key limitation of this method is the progressive degradation of fiber quality, particularly the reduction in fiber length and increased presence of short fibers, which may influence MPF release during end-use.

To examine how mechanical recycling alters fiber properties, the fiber length distribution of primary PES staple fibers and those subjected to one, two, and three cycles of mechanical shredding was analyzed (Tables 1 and S1). A marked reduction in mean fiber length was observed after the first recycling cycle, accompanied by a notable increase in short fiber count (SFC), defined as fibers shorter than 12.7 mm (i.e., 1/2 in.) according to industrial practice.^30^ Additional recycling cycles (rPES-2 and rPES-3) did not result in statistically significant further reductions in fiber length, suggesting a plateau effect after the initial degradation. However, the uniformity index continued to decline, indicating increasing heterogeneity in fiber length distribution.

**Table 1:** Properties of Fibers, Yarns, and Knitted Fabrics Produced from Primary Polyester (PES) and PES Containing 30% Mechanically Recycled Fibers after One, Two, and Three Recycling Cycles^a^

Test parameter		PES	rPES-1	rPES-2	rPES-3
Fiber length	mean length (mm)	32.4 ± 1.3	24.3 ± 1.2	24.7 ± 1.2	23.9 ± 0.9
	short fiber count (%)	5.2 ± 0.6	11.0 ± 1.4	10.5 ± 1.3	11.4 ± 1.1
	uniformity index (−)	90.8 ± 2.7	78.2 ± 1.5	78.6 ± 1.5	76.7 ± 3.3
Fiber loss during processing	sliver weight after drafting (g)	78.6 ± 0.4	78.1 ± 0.8	77.6 ± 1.3	78.4 ± 0.2
	loss of material during carding and drafting (%)	1.8	2.4	3.0	2.0
	linear density of single yarn (tex)	38	33	26	28
	loss of material during yarn spinning (%)		8	17	14
	fabric weight (g/m^2^)	400	340	310	360
Yarn hairiness	linear density of 2-ply yarn (tex)	77	65	51	56
	hairiness (−)	10.0 ± 2.1	11.9 ± 3.1	12.2 ± 2.8	12.5 ± 3.1
Pilling grade	Martindale after 7000 cycles	3	2–3	2–3	2–3
	ICI pilling box after 14,400 cycles	2–3	3–4	3	2

a Fiber length (*n* = 10), sliver weight (*n* ≥ 4) and yarn hairiness (*n* = 3) are reported as mean ± standard deviation, with corresponding raw data provided in the Supporting Information (Tables S1–S3). All other values are calculated quantities. Pilling performance was evaluated after wear simulation using a grading scale of 1–5, where 5 indicates no visible surface changes.

The observed decrease in fiber length following mechanical recycling is consistent with findings reported in previous studies.^31^ Prior research suggests that fiber length retention can be enhanced by applying lubricants, such as polyethylene glycol, to the fabric prior to the opening stage.^15^ Additionally, the orientation in which the fabric is fed into the opener has been shown to significantly influence both the degree of opening and the resulting fiber length.^29^ In this study, no lubricants were used, and the fabric was introduced into the edge opener in random orientations to simulate current industrial practices.

The resulting reduction in fiber length and increase in short fiber content (SFC) are known to influence processability and product quality and may also play a critical role in microplastic fiber (MPF) release during fabric use and care. To assess the intermediate processing impact, the weight of the drafted sliver and the linear density of the resulting yarn were measured. As shown in Tables 1 and S2, material loss during the carding and drafting phases remained relatively stable across all recycling rounds. However, a gradual increase in material loss was recorded during the fiber spinning stage with each successive recycling cycle. This was further substantiated by a concurrent decrease in yarn linear density, indicating that fiber loss not only occurred during processing but also affected the structural properties of the yarn. Interestingly, rPES-3 exhibited a slightly higher linear density than rPES-2, deviating from the expected trend. This variation is likely attributable to improved fiber cohesion under the higher humidity conditions used for processing rPES-3. The approximately 15% reduction in yarn linear density per recycling round is also reflected in the final fabric weights after knitting and prewashing. Fabrics produced from mechanically recycled fibers consistently exhibited lower weights than those made from the reference PES fabric.

Contrary to common assumptions that mechanically recycled fibers reduce yarn strength due to fiber degradation,^21,31,32^ the tenacity and elongation at break of the yarns in this study were not significantly affected by the incorporation of 30% mechanically recycled PES fibers, as shown in Figure 2 and Table S4. While mechanical recycling typically results in shorter and more damaged fibers, the production process appears to have filtered out a substantial portion of these low-quality fibers.

**Figure 2:**
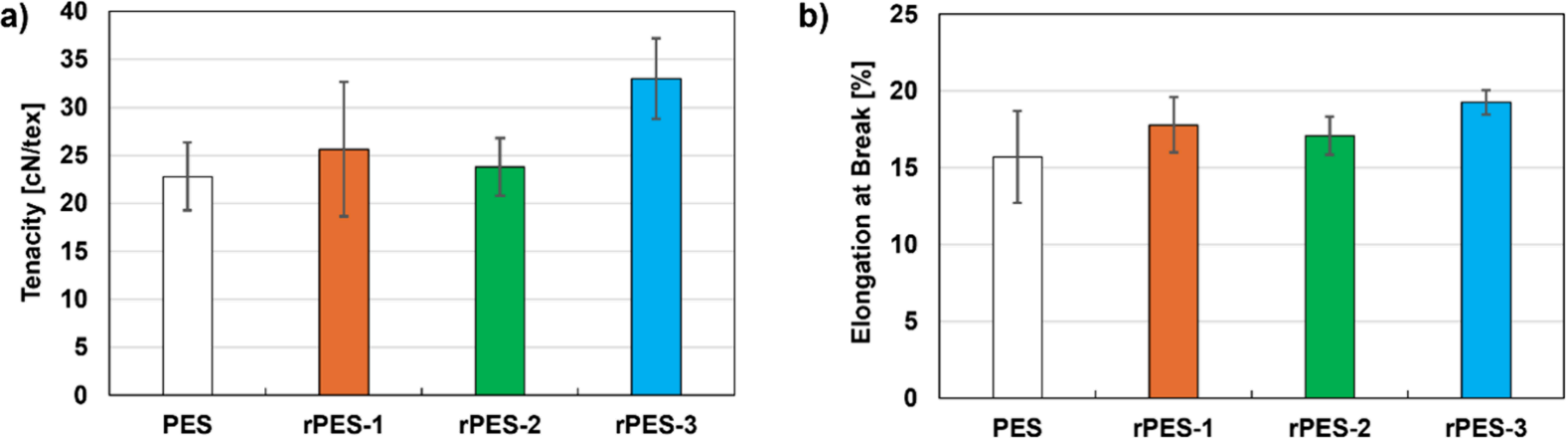
(a) Tenacity of PES yarn and PES yarns containing 30% mechanically recycled fibers after one (rPES-1) and two (rPES-2) recycling cycles. (b) Elongation at break for the same yarns (*n* = 10).

The observed decrease in yarn linear density is likely attributable to the preferential loss of short fibers during carding and spinning. These fibers, while present in the feedstock, contribute minimally to tensile strength and are more prone to removal during processing. As a result, the final yarns were composed primarily of longer, load-bearing fibers, which are more effective at transferring stress and maintaining structural integrity. This compositional shift likely contributed to the unexpected retention and slight increase in tensile strength despite a reduction in total fiber content.

These findings suggest that the selective retention of higher-quality fibers during spinning may counterbalance some of the adverse effects of recycling. However, this effect may be limited to moderate levels of recycled content. Higher incorporation rates or additional recycling cycles may still pose risks to mechanical performance, which warrants further investigation.

Yarn hairiness was evaluated using a yarn hairiness tester, and the results are summarized in Table 1 and S3. As expected, all yarns exhibited protruding fiber ends on their surfaces, regardless of the recycling stage. The yarn produced from primary PES showed the lowest level of hairiness, while yarns containing mechanically recycled fibers displayed a progressive increase in hairiness with each additional recycling cycle. This trend reflects a gradual deterioration in fiber length and a decline in structural uniformity as the number of recycling cycles increases, consistent with findings reported in previous studies.^31^

Although the mean fiber length and uniformity index of rPES-2 appear marginally higher than those of rPES-1 and rPES-3, statistical analysis (one-way ANOVA, *p* > 0.05) confirmed that these variations are not significant. The slightly higher fiber loss observed during carding and spinning for rPES-2 (Table 1) indicates that the material entering the second recycling cycle contained a larger proportion of weakened or brittle fibers, as well as potentially more unopened fiber bundles, which were more prone to breakage or rejection during processing and therefore removed as waste. This interpretation is supported by the reduced yarn linear density observed for rPES-2 (Table 1), which reflects a lower retained fiber mass. The lack of statistically significant differences in yarn hairiness further supports this conclusion and indicates that the variations among the recycled samples reflect normal process variability rather than a systematic effect of the recycling stage. Overall, the results demonstrate that repeated mechanical recycling under the conditions of this study did not significantly alter fiber or yarn performance, and the fluctuations observed between cycles appear to reflect normal process variability, with no statistically significant indication of progressive structural deterioration.

### Fiber Fragment Shedding during Wear Simulation

Microplastic fiber (MPF) shedding under simulated wear conditions was evaluated using the Martindale abrasion test to examine fiber release dynamics during extended mechanical stress. As shown in Figure 3a and Table S5 the highest MPF release occurred during the initial rubbing cycles. For primary PES and rPES-3, the greatest number of MPFs was collected within the first 1,000 cycles, while for rPES-1 and rPES-2, peak shedding occurred within 2,000 cycles. This pattern suggests that early stage wear primarily dislodges loosely bound or structurally compromised surface fibers, which are more prevalent in fabrics containing mechanically recycled fibers.

**Figure 3:**
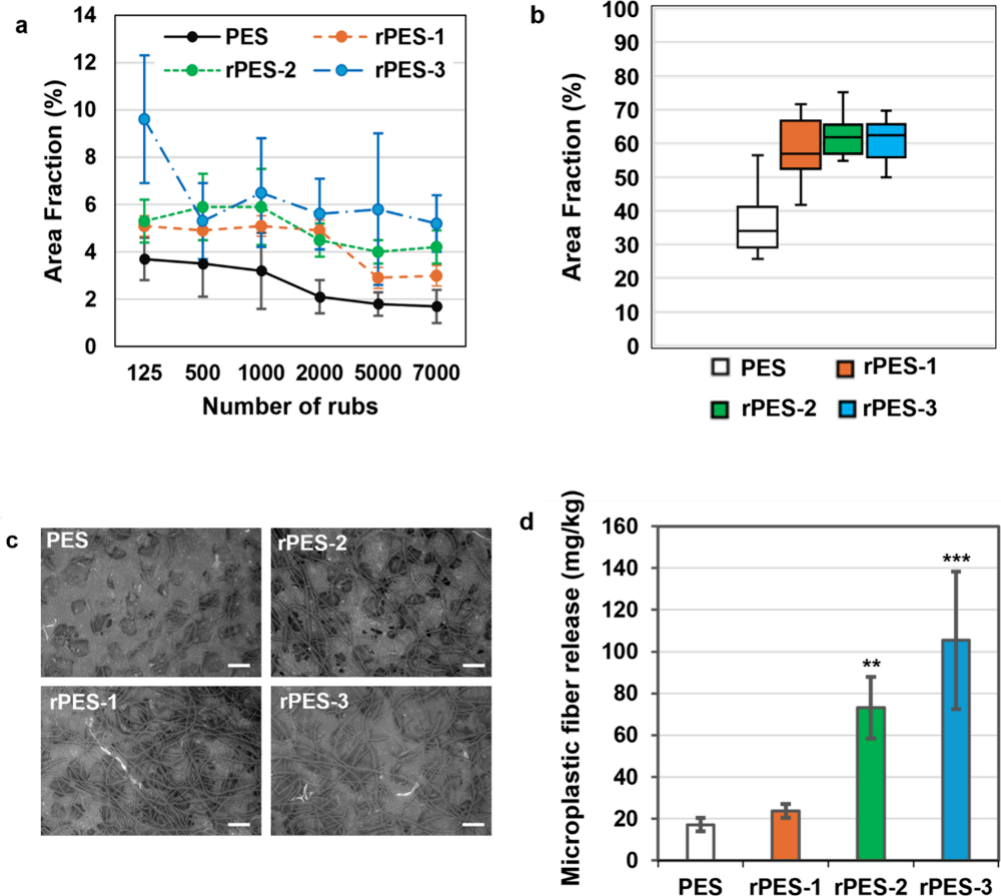
Microplastic fiber (MPF) release during simulated wear and laundering from fabrics produced from primary polyester (PES) yarn and PES yarn containing 30% mechanically recycled fibers after one, two, and three recycling cycles (rPES-1, rPES-2, and rPES-3). MPFs generated during wear were collected on carbon tape, and their area fraction (surface area occupied by MPFs) was quantified. (a) MPF release during Martindale testing at different rubbing intervals (*n* = 9). (b) Box plot of MPF area fraction obtained from the ICI pilling box test (*n* = 15). (c) Representative high-magnification SEM images from the ICI pilling box test (scale bar = 200 μm). (d) MPF release during laundering, tested following ISO 4484–1:2023. Data are expressed as MPF release (mg/kg fabric), with error bars representing standard deviation (*n* = 4). Asterisks indicate significant differences compared with PES based on Tukey’s HSD test (*p* < 0.01 for rPES-2; *p* < 0.001 for rPES-3). No significant differences were observed between PES and rPES-1 or between rPES-2 and rPES-3 (*p* > 0.05).

These observations align with the findings of Cai et al.,^33^ who attributed MPF generation directly to surface abrasion during Martindale testing of polyester textiles. As the number of rubbing cycles increased, MPF release declined, indicating that most easily detachable fibers are shed early in the abrasion process. This trend is further supported by Tong et al.^34^ who observed a similar release profile for both micro- and nanoplastic fragments during abrasion and washing of polyester fabrics.

Overall, fabrics containing mechanically recycled fibers (rPES-1, rPES-2, rPES-3) consistently exhibited higher MPF shedding than those made from primary PES. Additionally, visible accumulation of fiber dust near the metallic edges of the Martindale tester suggests a potential for airborne MPF emissions under prolonged abrasion. This represents an environmental pathway that remains relatively understudied but may have important implications for fiber dispersion in indoor and occupational settings.

To complement the Martindale test, the ICI Pilling Box was used to assess fiber fragmentation under controlled, localized abrasion. The results, presented in Figure 3(b-c) and Table S6, revealed increased MPF shedding in fabrics containing mechanically recycled fibers compared to primary PES. A progressive increase in shedding was observed across recycling cycles (rPES-1, rPES-2, rPES-3), reflecting the cumulative weakening of fiber structure. Statistical analysis confirmed these trends: Tukey’s HSD posthoc test indicated that primary PES had a significantly lower mean MPF area fraction than all recycled samples. Moreover, the SEM images confirm this pattern of fiber increase with recycling cycles. From a morphological point of view, there is no marked difference between the fibers in the different cycles. However, no significant differences were found among the recycled variants, suggesting a plateau effect after the first recycling cycle. This finding is consistent with earlier results indicating that most of the structural degradation, and the associated fiber shedding potential, occurs during the initial mechanical recycling stage. Novotná et al.^7^ similarly observed that abrasion plays a key role in MPF release from polyester fleece, complementing the shedding caused by washing and drying. In this context, our findings reinforce the importance of fiber fragmentation, rather than solely dislodged surface debris, as a primary mechanism of MPF generation in mechanically recycled textiles.

### Comparison between Pilling and MPF Shedding

The pilling grades of the fabric samples were evaluated following both the ICI Pilling Box and Martindale tests, with results summarized in Table 1. Overall, the fabrics exhibited pilling resistance values that fall within the typical range expected for knitted polyester textiles. No clear correlation was observed between a fabric’s tendency to form pills and its MPF shedding behavior. Although minor differences in pilling grades were recorded across samples, these did not align consistently with MPF release trends. For example, fabrics made from primary PES did not exhibit markedly worse pilling performance than recycled variants yet consistently released fewer MPFs (Figure 3a-b). In contrast, fabrics incorporating mechanically recycled fibers showed slightly higher pilling grades in some cases but exhibited significantly greater MPF shedding. These findings suggest that pilling resistance, as traditionally assessed, is not a reliable indicator of environmental performance in terms of fiber release.

This apparent disconnect may be explained by differences in fiber strength. Pills formed from stronger, cohesive fibers tend to remain attached to the fabric surface, while weaker fibers, such as those found in mechanically recycled materials, are more prone to detachment, directly contributing to MPF shedding. Microscopic analysis of the carbon tape confirmed a higher incidence of pill detachment in fabrics made with recycled fibers, supporting this interpretation.

Pills can negatively affect the visual appearance of fabrics, which is often perceived as a quality issue by consumers. As a result, pilling resistance remains an important parameter in textile performance assessment.^6^ Traditional pilling grade evaluation relies on subjective visual inspection, introducing potential evaluator bias and inconsistency.^35^ Although emerging methods based on image analysis and deep learning show promise in improving objectivity,^36^ this study relied on standard visual grading protocols. Therefore, the possibility of grading bias cannot be excluded. Furthermore, it is established that fabrics made from high-twist filament yarns typically exhibit greater resistance to both pilling and MPF release^37^ reinforcing the importance of fiber cohesion and yarn structure. Taking together, these findings indicate that evaluating pilling propensity alone is insufficient to predict a fabric’s MPF shedding behavior. The relationship between surface wear, fiber strength, and MPF release is more complex and requires separate, targeted testing for reliable environmental assessment.

### Microplastic Fiber Shedding during Laundry

Fiber release during laundering was evaluated in accordance with SS-EN-ISO 4484–1:2023, and the results are presented in Figure 3d and Tables S7 and S8. MPF shedding did not differ significantly between fabrics made from primary PES and those incorporating fibers subjected to one cycle of mechanical recycling (rPES-1). This result aligns with previous studies reporting that limited incorporation of recycled fibers does not substantially increase MPF shedding during laundering.^38,39^ In contrast, fabrics containing fibers that had undergone two and three recycling cycles (rPES-2 and rPES-3) released significantly more MPFs than PES (p = 0.004 and *p* < 0.001, respectively) and rPES-1 (p = 0.011 and *p* < 0.001, respectively). No significant difference was observed between rPES-2 and rPES-3 (p = 0.108). A one-way ANOVA followed by Tukey’s HSD test confirmed this distinction, indicating a statistically significant separation between lower-recycled and higher-recycled samples.

To place these differences into a practical context, we estimated the potential annual MPF release per person based on an average laundry volume of 260 kg/year (approximately two loads per week, each weighing 2.5 kg). Laundering fabrics made from primary PES would result in approximately 4.45 g MPF/year per person, whereas rPES-1, rPES-2, and rPES-3 fabrics would release about 6.19 g/year, 19.0 g/year, and 27.3 g/year, respectively. These estimates assume that all laundered fabrics are of the same composition and are intended solely to contextualize the relative magnitude of MPF emissions at different recycling stages (see Supporting Information Table S8 for calculations).

This finding is particularly important in the context of current testing standards. Laundering is currently the only condition for which standardized MPF shedding methods exist, as defined in AATCC TM212 and ISO 4484–1:2023. In contrast, no standardized methods are available for evaluating MPF shedding under dry-state conditions, such as those encountered during wear and abrasion, despite growing evidence of their contribution to environmental fiber release.

Increased variability in MPF shedding among the highly recycled samples also suggests growing heterogeneity in fiber structure. This interpretation is supported by a corresponding increase in yarn hairiness observed across recycling cycles, which likely contributes to elevated fiber fragmentation during laundering. Furthermore, differences in shedding behavior between laundering and wear simulation (Figures 3a-d) underscore the role of different mechanical stressors and highlight the importance of maintaining fiber integrity throughout the recycling process. Liu et al.^40^ similarly reported that fiber release under mechanical abrasion is substantially higher than under standard washing protocols, reinforcing the need to assess MPF shedding under both dry and wet conditions. These findings collectively highlight the susceptibility of synthetic fibers, particularly those weakened by repeated mechanical recycling, to fragmentation under frictional stress.

If MPF release had been assessed using laundering alone, the extent of shedding from highly recycled fibers may have been underestimated. These results emphasize the value of using multiple test methods and reinforce the need for standardized dry-state testing protocols to provide a more complete picture of a fabric’s environmental impact. Building on these findings, it is clear that addressing MPF release requires both accurate assessment and proactive mitigation strategies. These findings highlight the need for more systematic evaluation of MPF shedding in mechanically recycled textiles. As MPFs are known to act as carriers for hazardous substances, the increased release observed from highly recycled fabrics raises environmental concerns that go beyond fiber loss alone. Addressing this issue will require targeted efforts to mitigate shedding, including strategies such as improving fiber length retention during recycling, optimizing spinning conditions, and applying finishing treatments that enhance fiber cohesion.

## Implications for Future Research and Action

Recycling of polyester textiles offers clear environmental advantages compared to uncontrolled disposal practices, such as open dumping or landfilling, which contribute to persistent environmental pollution. However, our findings indicate that recycling history plays a critical role in microplastic fiber (MPF) shedding. Fabrics containing 30% recycled content from a single cycle (rPES-1) shed fibers at rates comparable to primary PES, whereas shedding increased substantially after the second and third recycling cycles.

Importantly, abrasion during wear generated higher fiber release than laundering, indicating that laundering-based tests alone may underestimate the true extent of MPF emissions. This reinforces the need for standardized methods that also capture dry-state shedding to more accurately reflect real-world textile use.

The results highlight a trade-off: while recycling contributes to circularity and reduces environmental burdens from waste disposal and primary fiber production, repeated mechanical recycling can compromise fiber integrity, leading to greater MPF release. Mitigation strategies should focus on enhancing fiber length retention during recycling, optimizing yarn and fabric construction, and applying surface treatments that improve cohesion. Although these measures may increase production costs, they represent necessary investments to ensure that textile recycling delivers net environmental benefits without exacerbating microplastic pollution.

Future research should quantify MPF emissions across full product lifecycles and explore fiber innovations designed to resist release, particularly in recycled materials. An outlook for recycled fibers that are intrinsically less prone to MPF shedding should also be considered. A balanced approach that advances textile recycling while minimizing MPF pollution is essential for achieving both circular economy and environmental protection goals.

## Supplementary Material



## Data Availability

High-resolution images are available from the authors upon reasonable request.

## Notes

Supplementary figures and tables including photographs illustrating key stages of yarn production and mechanical recycling (Figure S1), an illustration of the optical microscopy image analysis workflow used to calculate the area fraction (Figure S2), and tables with data on fiber length after shredding knitted fabrics (Table S1), sliver weight after drafting (Table S2), yarn hairiness (Table S3), tenacity and elongation at break (Table S4), area fraction images from Martindale (Table S5) and ICI box pilling testing (Table S6), and fiber loss calculations (Table S7), including conversion of mean fiber loss during washing (Table S8) (PDF)
