# Supplementary material for: Mechanically Recycled Textiles: A Source of Microplastic Fiber Emissions
Source: Environ Sci Technol. 2026 Jan 7;60(2):1810–8. doi: 10.1021/acs.est.5c14973 (PMC12825150; doi:10.1021/acs.est.5c14973)
Supplement: Supplementary file 1 [file es5c14973_si_001.pdf]

Mechanically Recycled Textiles - A source of Microplastic Fiber Emissions

Maria Persson[1*], Juliana Aristéia de Lima[2,3], Nawar Kadi[1], Nils-Krister Persson[1,4]

[1] The Swedish School of Textiles, Faculty of Textiles, Engineering and Business, University of Borås, 501 90 Borås, Sweden

[2] Swedish Centre of Resource Recovery, Faculty of Textiles, Engineering and Business, University of Borås, 501 90 Borås, Sweden

[3] Department of Polymer, Fiber and Composite, RISE Research Institutes of Sweden, 504 62 Borås, Sweden

[4] Polymer E-textiles, The Swedish School of Textiles, Smart Textiles, Science Park Borås, University of Borås, 501 90 Borås, Sweden

*Corresponding author: maria.persson@hb.se

Supplementary file information: 25 pages, 2 figures and 8 tables.

Figure S1: Photographs illustrating key stages of yarn production and mechanical recycling:

Figure S2: Illustration of the process analyzing the optical microscope image to calculate the area fraction (%).

**Table of contents for supplementary tables**	
**Table**	**Title**
S1	Fiber length after shredding the knitted fabrics
S2	Sliver weight after drafting
S3	Yarn hairiness
S4	Tenacity and Elongation at Break
S5	Images used to estimate the area fraction after Martindale testing
S6	Images used to estimate the area fraction after ICI box pilling testing
S7	Determination of fiber loss from fabric during washing
S8	Conversion of mean fiber loss from fabrics during washing

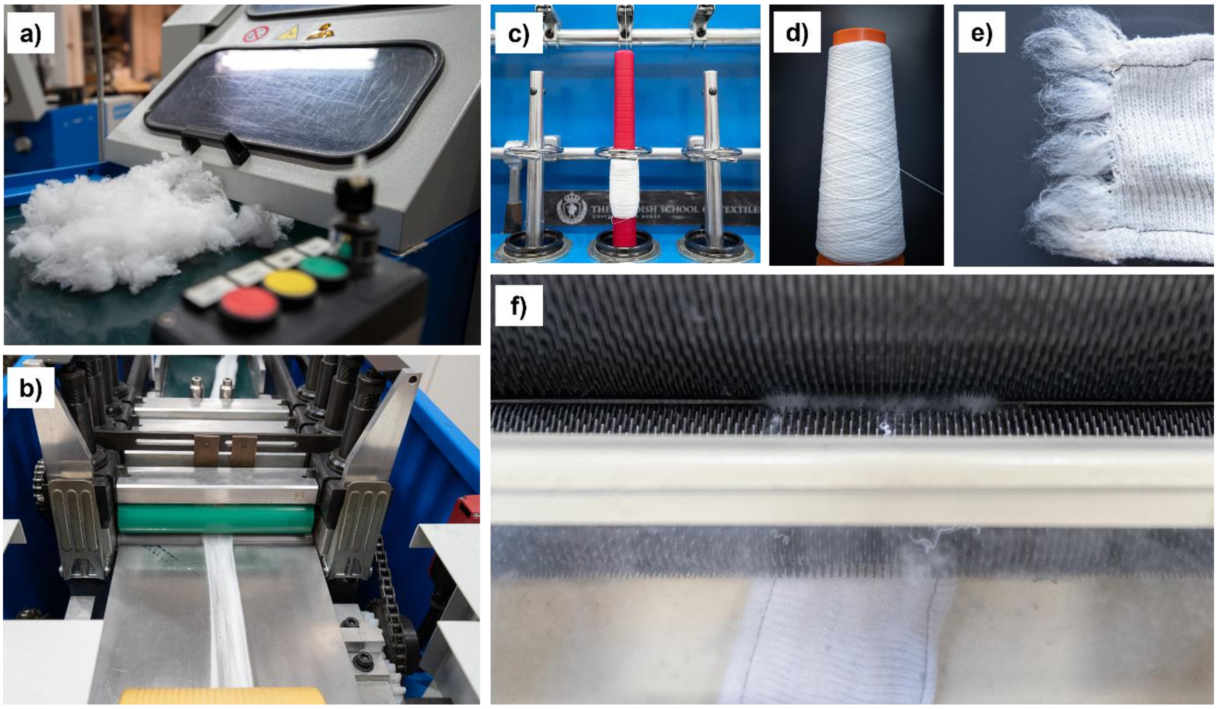

Figure S1. Photographs illustrating key stages of yarn production and mechanical recycling: (a) staple fiber prior to carding, (b) drafting process (c) ring spinning of single yarn (d) twisted ply yarn (e) knitted fabric edge after exposure to the shredding machine, and (f) knitted fabric undergoing shredding

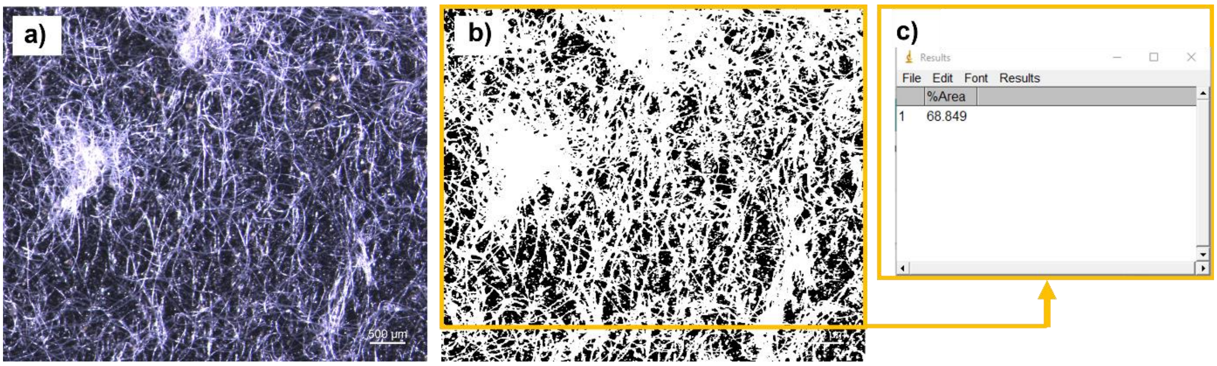

Figure S2. Illustration of the process analyzing the optical microscope image to calculate the area fraction [%]. (a) Original microscope image, b) Image converted to black and white (binary mode) c) ImageJ output showing the calculated area fraction of the fibers.

Table S1: Fiber length after shredding the knitted fabrics

Sample	Replicate	Mean Length (mm)	Short Fiber count (SFC)	Uniformity index (UI)
PES	1	34.3	4.51	93.8
	2	34.2	4.54	93.5
	3	32.8	5.06	93.4
	4	32.4	5.22	90.2
	5	32.4	5.21	91.4
	6	32.6	5.12	91.2
	7	32.6	5.15	91.5
	8	32.2	5.31	89.0
	9	29.9	6.39	85.4
	10	31.0	5.83	88.1
PES-1	1	26.0	9.19	80.4
	2	24.1	11.2	78.3
	3	24.6	10.5	77.9
	4	24.4	10.8	78.5
	5	24.1	11.2	78.1
	6	22.6	13.1	76.7
	7	26.4	8.82	80.8
	8	23.4	12.0	76.9
	9	24.6	10.6	78.2
	10	22.8	12.8	76.1
PES -2	1	23.8	11.5	78.4
	2	23.7	11.6	76.6
	3	23.1	12.4	77.3
	4	23.6	11.7	76.9
	5	26.1	9.03	79.5
	6	24.8	10.3	78.4
	7	24.5	10.6	77.9
	8	25.4	9.7	80.1
	9	25.6	9.55	81.4
	10	26.7	8.59	79.7
PES-3	1	23.8	11.5	78.9
	2	23.4	11.9	77.1

	3	24.2	11.0	77.6
	4	25.6	9.50	79.5
	5	24.2	11,1	76.8
	6	23.5	11.9	77.3
	7	22.7	12.9	69.9
	8	22.6	12.9	72.0
	9	23.9	11.4	76.7
	10	25.1	9.9	81.5

Table S2. Sliver weight after drafting

Sample	Replicate	Weight (g)
PES	1	78.6
	2	78.2
	3	79.0
	4	78.1
	5	78.6
	6	79.4
	7	78.5
	8	79.1
PES-1	1	78.7
	2	78.2
	3	78.4
	4	76.9
PES-2	1	78.3
	2	79.0
	3	76.0
	4	77.2
PES-3	1	78.7
	2	78.2
	3	78.4
	4	78.3

Table S3: Yarn Hairiness

Sample	Replicate (Hairiness, H)		
	1	2	3
PES	9.89	9.87	10.20
rPES-1	12.51	11.16	11.96
rPES-2	12.75	11.90	12.03
rPES-3	11.80	12.91	12.75

Table S4. Tenacity and Elongation at Break

Sample	Replicate	Tenacity (cN/tex)	Elongation at Break (%)
PES	1	24.5	16.1
	2	21.1	13.6
	3	26.9	18.6
	4	28.8	20.5
	5	24.3	13.6
	6	19.3	16.3
	7	23.4	13.7
	8	17.9	15.1
	9	22.8	18.7
	10	18.9	10.6
rPES-1	1	25.3	18.6
	2	27.9	18.0
	3	31.4	18.7
	4	24.0	17.5
	5	19.9	17.6
	6	15.3	15.9
	7	33.7	20.5
	8	36.8	19.9
	9	17.2	14.5
	10	24.9	17.4
rPES-2	1	26.7	17.8
	2	24.8	17.8
	3	23.3	16.9
	4	18.2	15.4

	5	25.3	17.1
	6	22.0	15.7
	7	24.4	18.3
	8	26.9	18.7
	9	21.8	16.7
	10	28.8	18.6
rPES-3	1	29.7	18.7
	2	32.5	18.5
	3	33.8	20.1
	4	31.7	18.1
	5	41.0	20.7
	6	34.9	19.6
	7	34.2	19.8
	8	39.2	19.8
	9	28.9	19.0
	10	29.3	18.5

Table S5: Images used to estimate the area fraction after Martindale testing

PES				
125	Original image	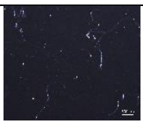	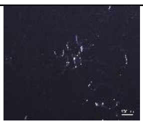	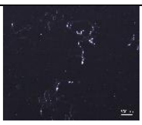
	Binary image	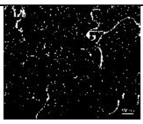	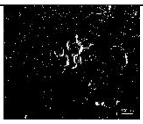	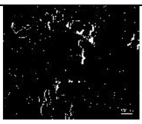
	Area Fraction (%)	4.0	3.0	2.7
	Original image	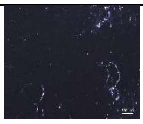	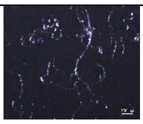	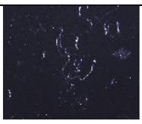
	Binary image	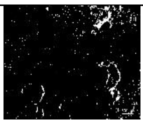	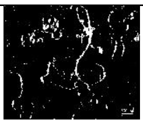	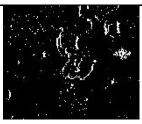
	Area fraction (%)	3.7	5.5	3.9
	Original image	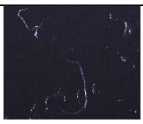	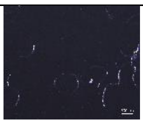	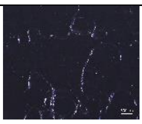

	Binary image	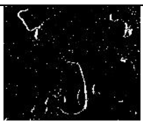	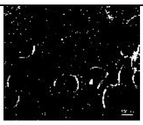	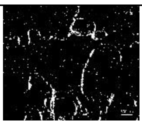
	Area Fraction (%)	2.8	3.4	4.4
500	Original image	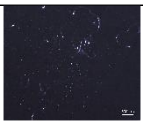	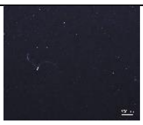	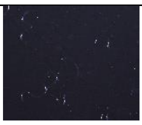
	Binary image	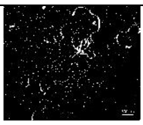	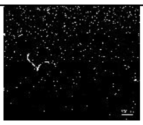	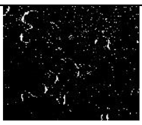
	Area Fraction (%)	2.3	3.3	2.8
	Original image	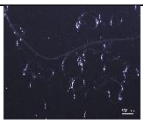	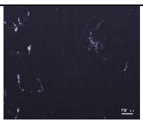	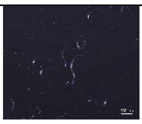
	Binary image	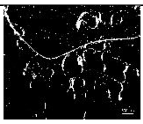	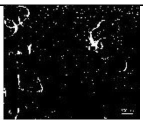	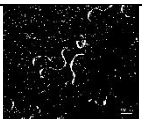
	Area Fraction (%)	6.3	3.4	3.8
	Original image	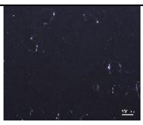	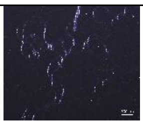	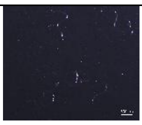
	Binary image	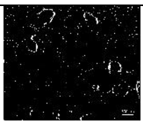	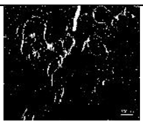	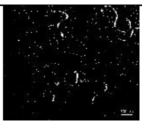
	Area Fraction (%)	2.3	4.7	2.0
1000	Original image	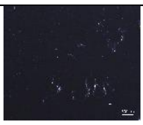	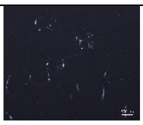	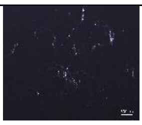
	Binary image	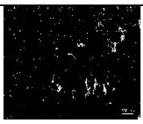	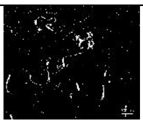	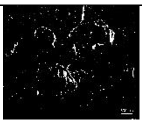
	Area Fraction (%)	2.3	2.5	2.7
	Original image	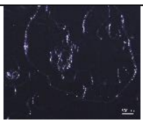	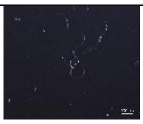	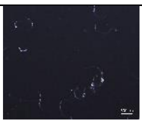
	Binary image	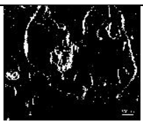	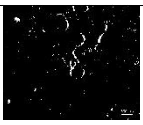	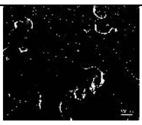
	Area Fraction (%)	6.7	1.9	2.4

	Original image	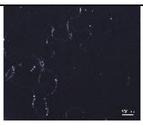	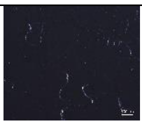	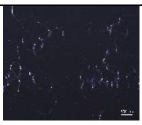
	Binary image	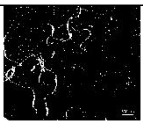	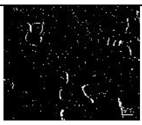	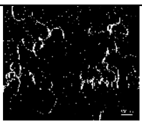
	Area Fraction (%)	3.4	2.4	4.9
2000	Original image	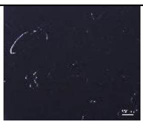	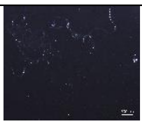	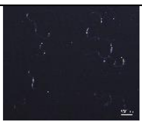
	Binary image	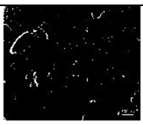	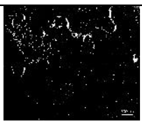	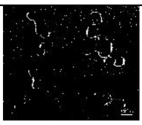
	Area Fraction (%)	1.6	2.9	2.1
	Original image	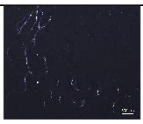	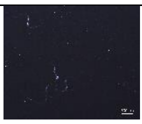	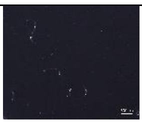
	Binary image	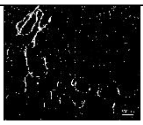	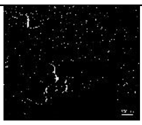	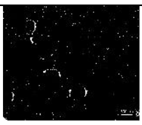
	Area Fraction (%)	3.5	1.6	1.1
	Original image	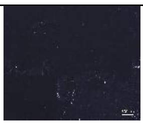	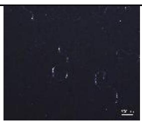	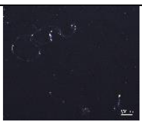
	Binary image	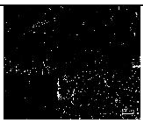	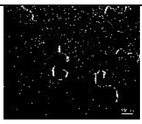	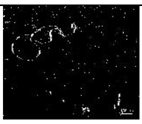
	Area Fraction (%)	2.0	2.5	1.7
5000	Original image	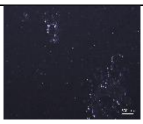	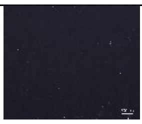	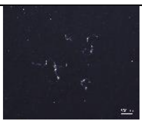
	Binary image	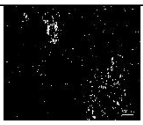	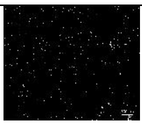	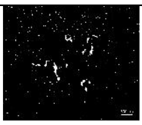
	Area Fraction (%)	1.9	0.8	1.8
	Original image	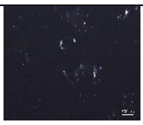	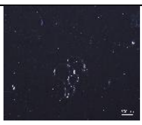	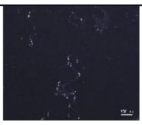

	Binary image	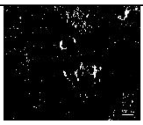	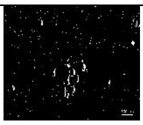	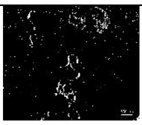
	Area Fraction (%)	2.4	1.7	2.4
	Original image	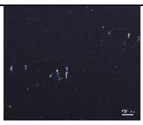	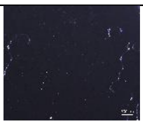	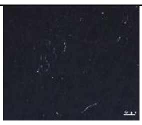
	Binary image	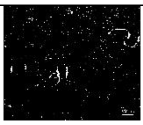	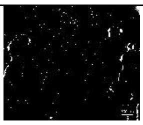	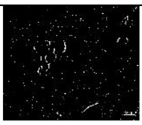
	Area Fraction (%)	2.0	1.6	1.6
7000	Original image	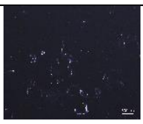	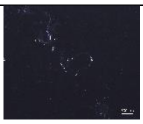	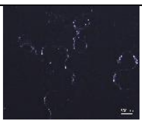
	Binary image	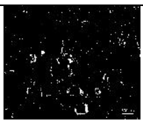	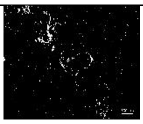	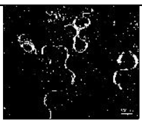
	Area Fraction (%)	2.1	2.0	2.4
	Original image	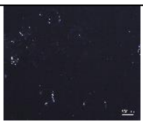	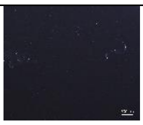	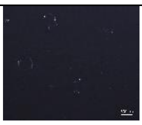
	Binary image	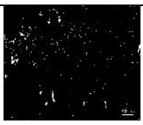	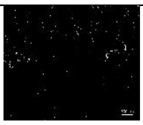	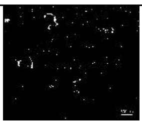
	Area Fraction (%)	1.5	0.6	1.0
	Original image	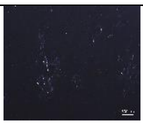	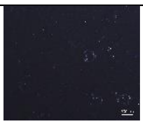	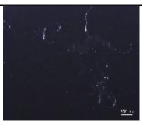
	Binary image	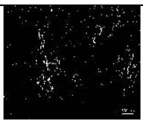	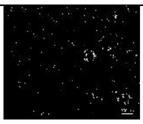	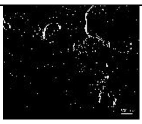
	Area Fraction (%)	2.1	1.0	2.3
PES-1				
	Original image	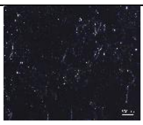	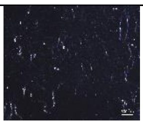	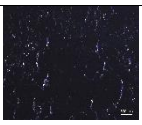
	Binary image	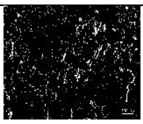	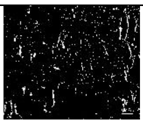	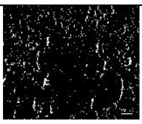
	Area Fraction (%)	5.6	4.9	5.1

	Original image	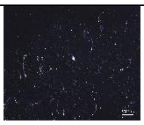	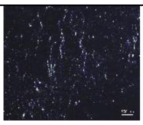	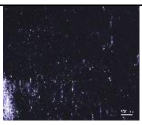
	Binary image	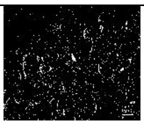	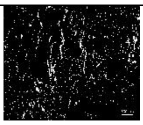	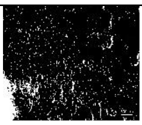
	Area Fraction (%)	4.6	6.1	7.9
	Original image	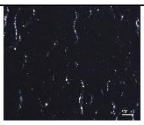	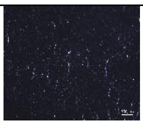	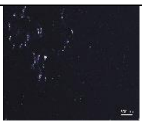
	Binary image	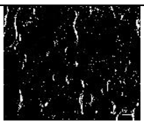	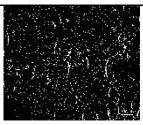	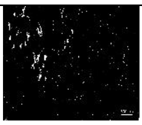
	Area Fraction (%)	4.4	5.8	1.9
500	Original image	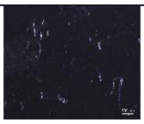	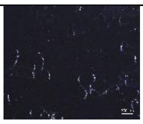	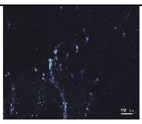
	Binary image	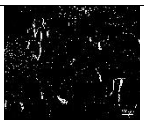	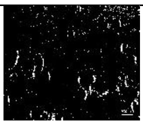	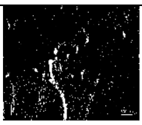
	Area Fraction (%)	3.5	3.4	4.3
	Original image	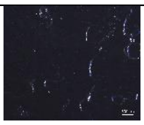	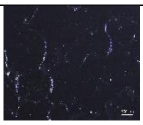	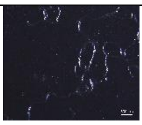
	Binary image	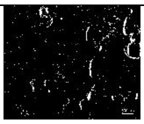	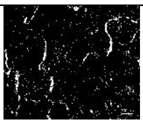	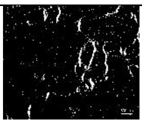
	Area Fraction (%)	3.5	4.5	5.0
	Original image	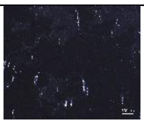	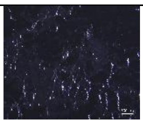	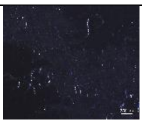
	Binary image	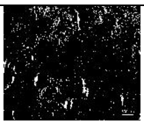	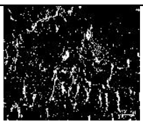	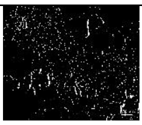
	Area Fraction (%)	5.6	9.8	4.4
1000	Original image	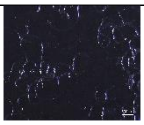	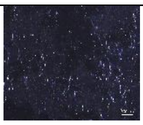	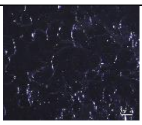

	Binary image	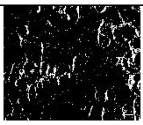	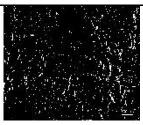	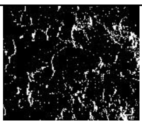
	Area Fraction (%)	8.0	4.9	8.5
	Original image	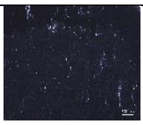	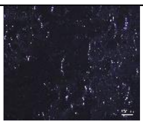	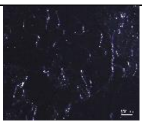
	Binary image	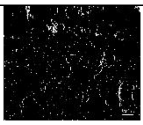	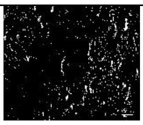	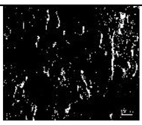
	Area Fraction (%)	3.2	5.0	5.2
	Original image	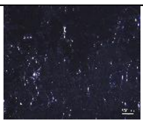	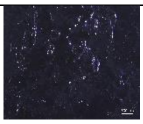	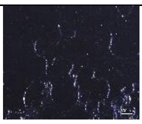
	Binary image	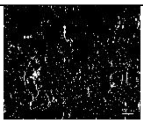	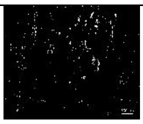	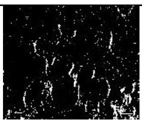
	Area Fraction (%)	5.1	1.6	4.6
2000	Original image	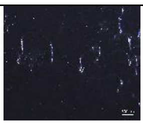	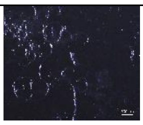	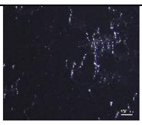
	Binary image	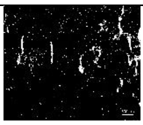	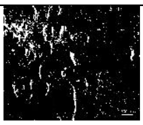	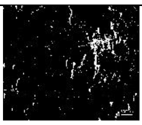
	Area Fraction (%)	4.9	7.5	4.6
	Original image	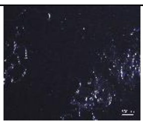	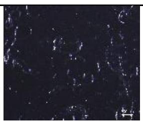	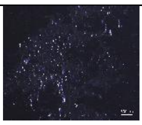
	Binary image	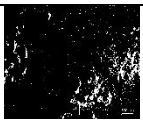	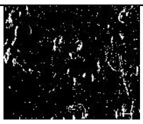	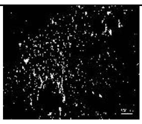
	Area Fraction (%)	6.2	5.4	4.2
	Original image	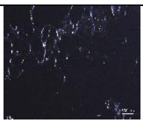	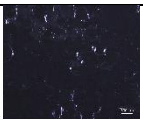	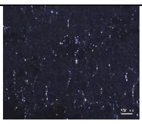
	Binary image	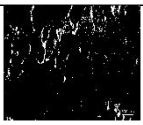	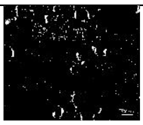	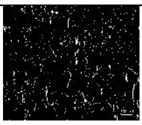
	Area Fraction (%)	4.3	2.8	3.8

5000	Original image	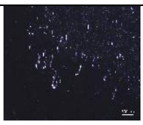	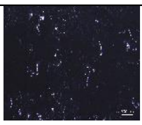	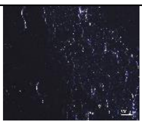
	Binary image	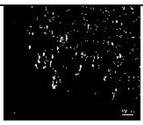	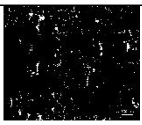	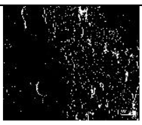
	Area Fraction (%)	2.4	2.9	5.0
	Original image	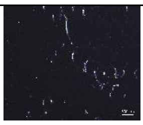	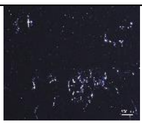	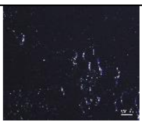
	Binary image	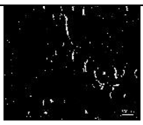	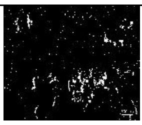	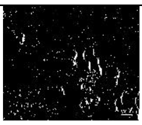
	Area Fraction (%)	2.0	3.7	3.4
	Original image	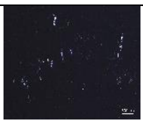	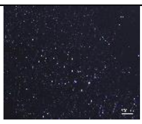	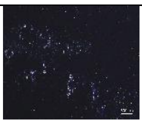
	Binary image	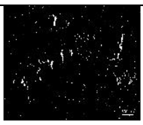	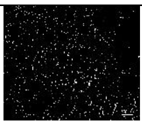	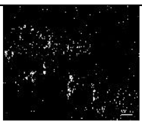
	Area Fraction (%)	1.8	2.7	2.2
7000	Original image	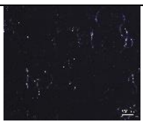	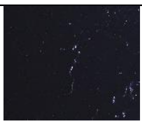	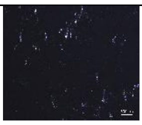
	Binary image	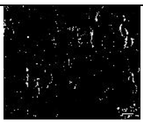	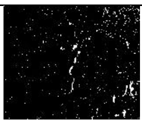	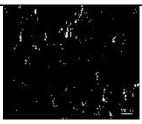
	Area Fraction (%)	2.2	2.7	1.6
	Original image	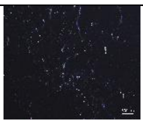	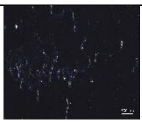	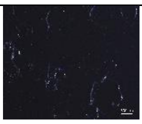
	Binary image	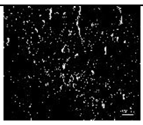	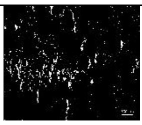	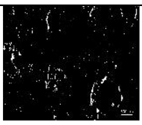
	Area Fraction (%)	4.1	3.8	2.2
	Original image	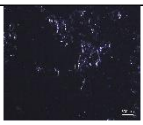	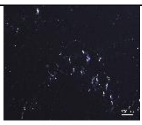	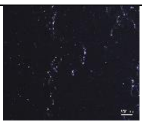

	Binary image	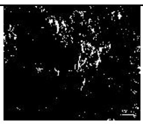	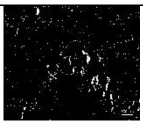	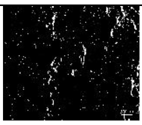
	Area Fraction (%)	4.2	2.8	3.6
rPES-2				
125	Original image	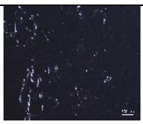	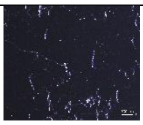	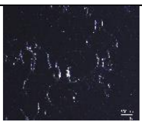
	Binary image	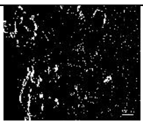	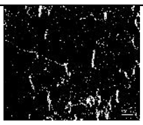	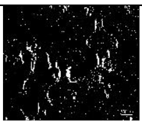
	Area Fraction (%)	5.0	5.0	4.6
	Original image	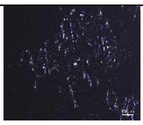	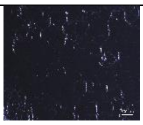	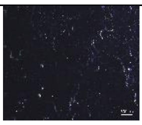
	Binary image	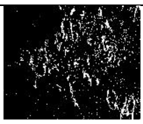	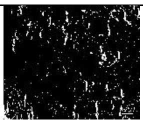	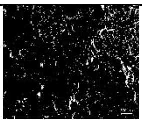
	Area Fraction (%)	8.2	5.4	6.5
	Original image	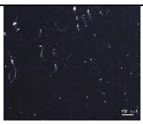	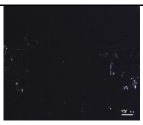	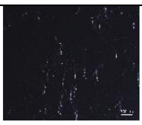
	Binary image	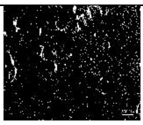	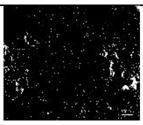	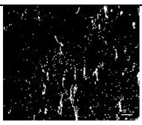
	Area Fraction (%)	5.4	3.3	4.4
500	Original image	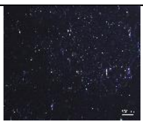	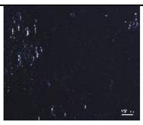	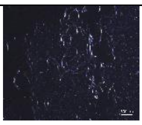
	Binary image	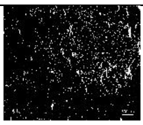	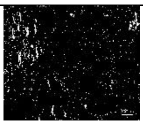	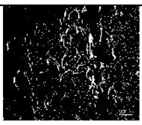
	Area Fraction (%)	6.5	4.3	7.9
	Original image	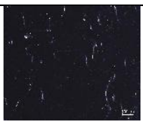	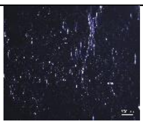	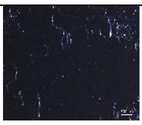
	Binary image	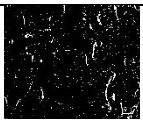	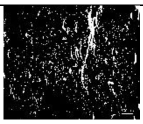	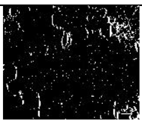
	Area Fraction (%)	4.4	9.5	4.9

	Original image	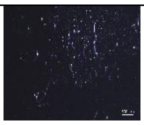	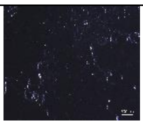	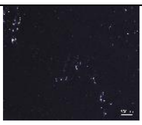
	Binary image	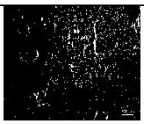	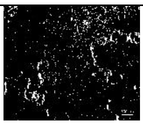	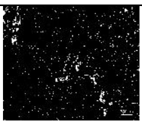
	Area Fraction (%)	5.3	6.1	3.9
1000	Original image	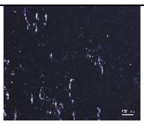	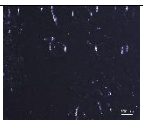	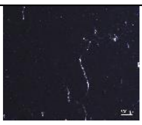
	Binary image	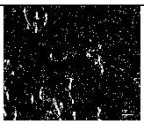	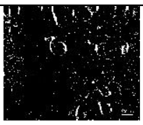	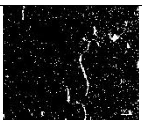
	Area Fraction (%)	5.1	4.6	4.4
	Original image	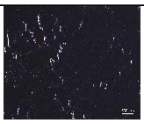	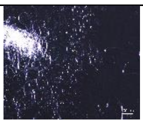	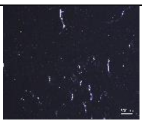
	Binary image	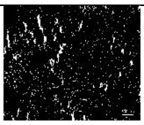	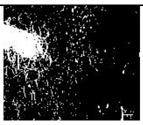	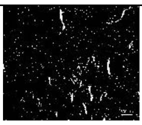
	Area Fraction (%)	5.9	13.7	3.4
	Original image	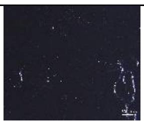	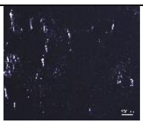	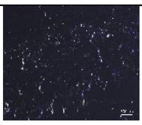
	Binary image	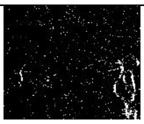	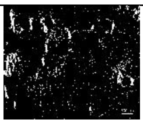	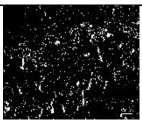
	Area Fraction (%)	3.1	6.6	6.2
2000	Original image	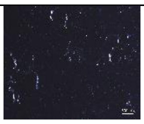	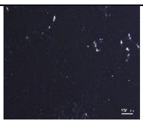	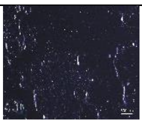
	Binary image	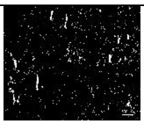	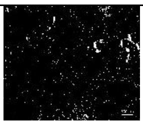	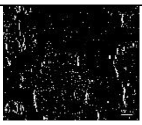
	Area Fraction (%)	3.6	2.7	4.3
	Original image	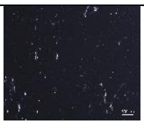	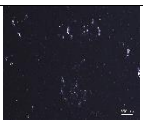	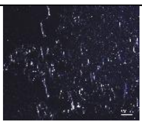

	Binary image	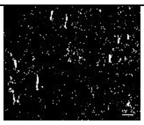	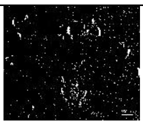	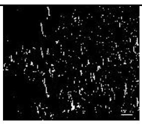
	Area Fraction (%)	4.4	3.3	4.0
	Original image	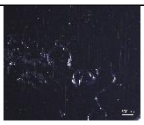	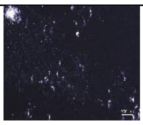	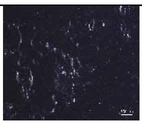
	Binary image	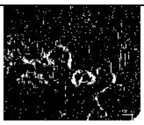	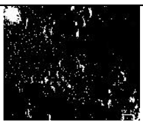	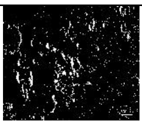
	Area Fraction (%)	7.3	5.2	6.0
5000	Original image	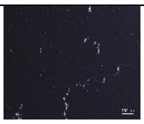	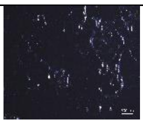	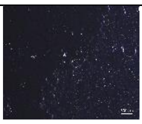
	Binary image	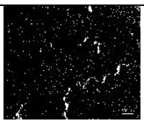	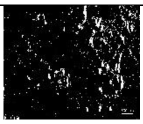	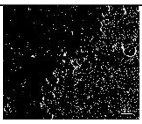
	Area Fraction (%)	3.1	6.2	5.9
	Original image	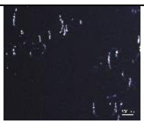	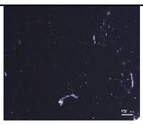	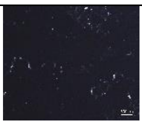
	Binary image	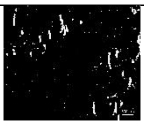	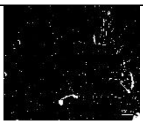	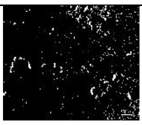
	Area Fraction (%)	3.2	2.2	3.8
	Original image	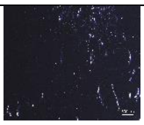	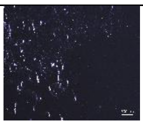	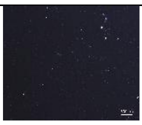
	Binary image	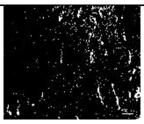	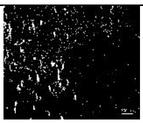	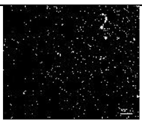
	Area Fraction (%)	4.8	4.7	2.4
7000	Original image	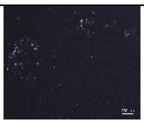	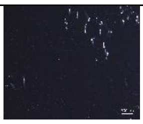	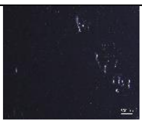
	Binary image	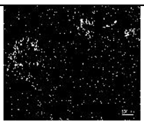	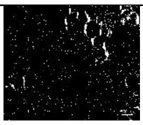	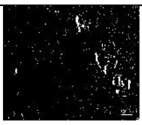
	Area Fraction (%)	3.3	4.2	2.7

	Original image	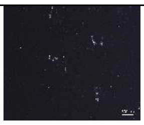	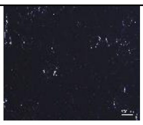	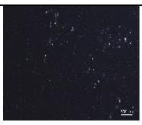
	Binary image	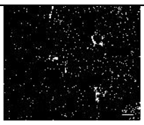	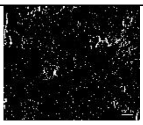	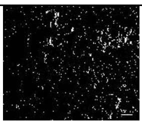
	Area Fraction (%)	2.9	4.3	3.7
	Original image	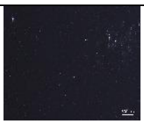	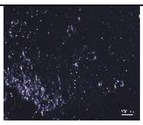	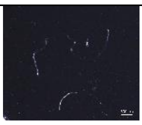
	Binary image	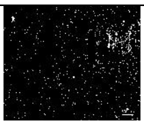	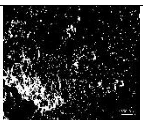	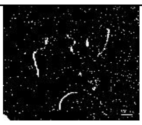
	Area Fraction (%)	3.3	10.6	3.2
**rPES-3**				
125	Original image	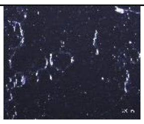	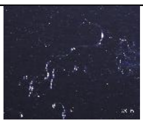	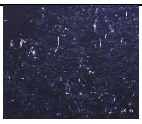
	Binary image	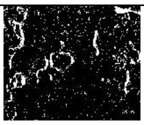	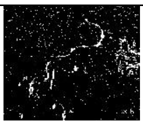	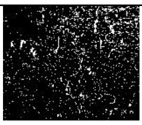
	Area Fraction (%)	9.5	5.5	9.6
	Original image	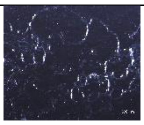	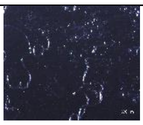	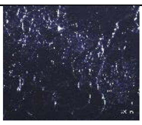
	Binary image	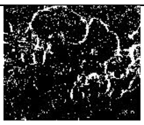	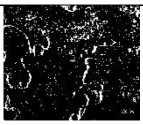	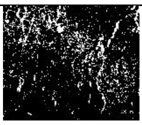
	Area Fraction (%)	13.7	9.0	13.6
	Original image	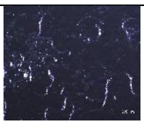	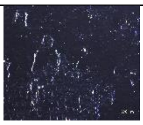	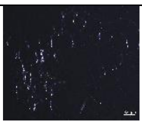
	Binary image	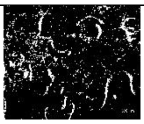	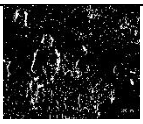	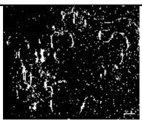
	Area Fraction (%)	10.2	7.6	7.9
500	Original image	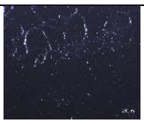	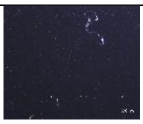	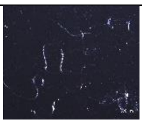

	Binary image	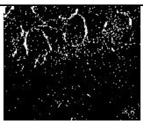	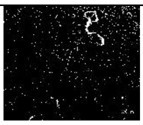	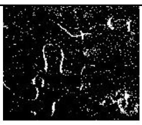
	Area Fraction (%)	6.1	3.3	6.8
	Original image	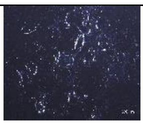	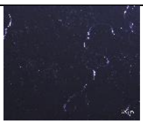	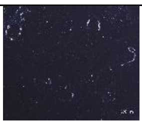
	Binary image	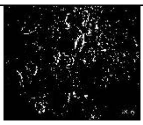	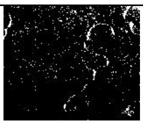	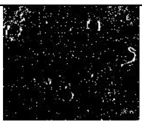
	Area Fraction (%)	4.5	4.4	4.1
	Original image	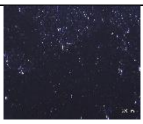	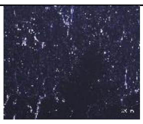	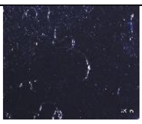
	Binary image	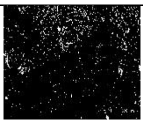	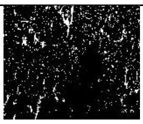	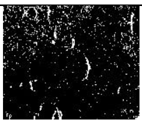
	Area Fraction (%)	5.1	8.3	6.4
1000	Original image	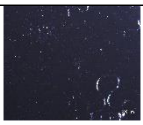	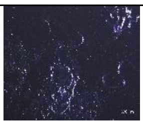	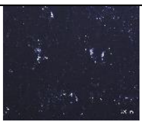
	Binary image	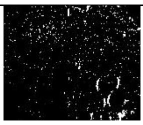	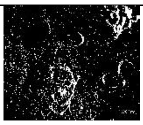	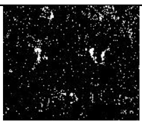
	Area Fraction (%)	3.8	8.9	5.3
	Original image	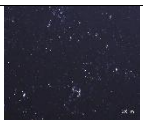	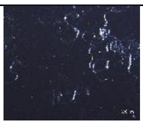	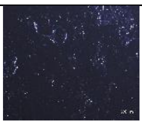
	Binary image	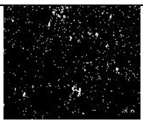	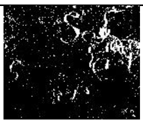	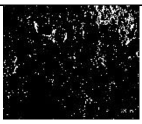
	Area Fraction (%)	3.5	8.9	5.5
	Original image	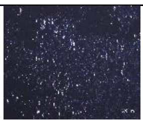	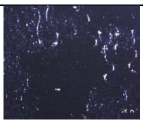	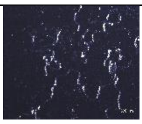
	Binary image	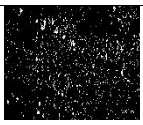	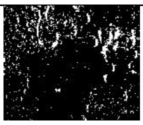	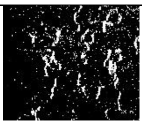
	Area Fraction (%)	6.5	9.7	9.6

2000	Original image	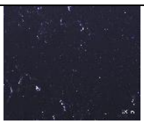	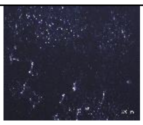	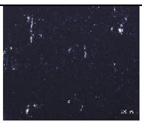
	Binary image	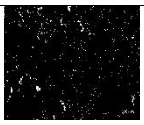	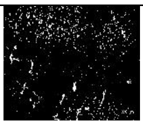	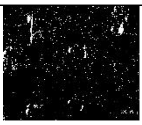
	Area Fraction (%)	2.2	4.8	4.0
	Original image	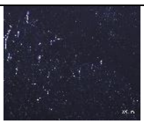	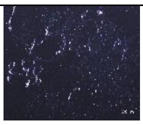	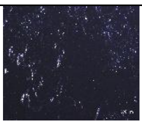
	Binary image	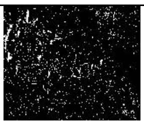	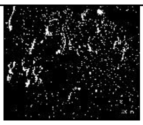	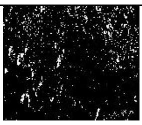
	Area Fraction (%)	6.3	6.9	6.3
	Original image	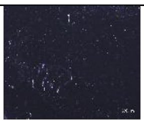	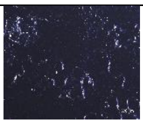	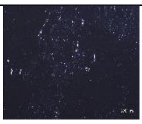
	Binary image	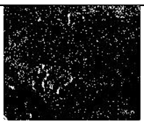	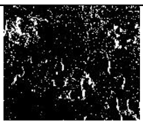	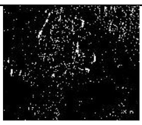
	Area Fraction (%)	5.6	9.0	5.2
5000	Original image	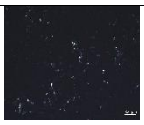	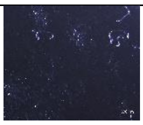	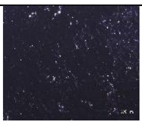
	Binary image	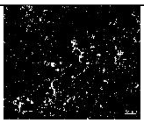	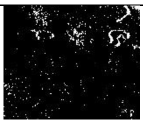	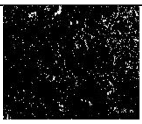
	Area Fraction (%)	4.1	3.9	4.5
	Original image	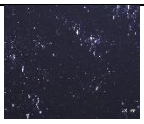	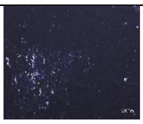	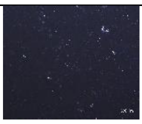
	Binary image	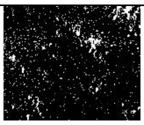	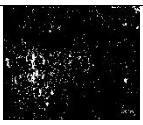	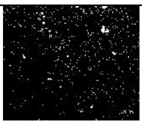
	Area Fraction (%)	7.3	4.3	3.2
	Original image	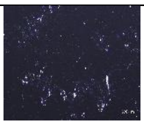	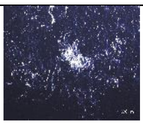	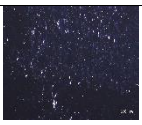

	Binary image	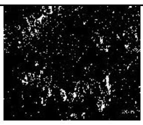	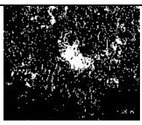	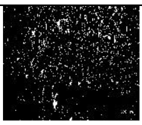
	Area Fraction (%)	5.7	13.2	6.9
7000	Original image	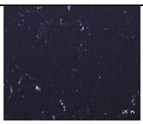	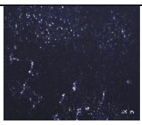	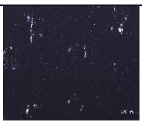
	Binary image	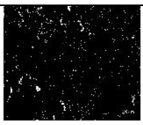	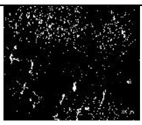	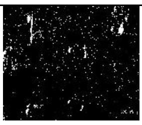
	Area Fraction (%)	8.5	4.6	3.5
	Original image	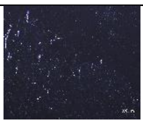	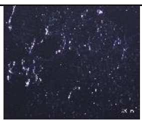	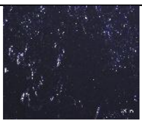
	Binary image	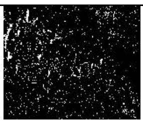	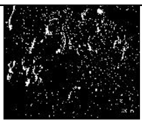	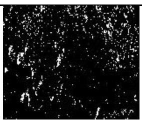
	Area Fraction (%)	3.4	3.8	5.0
	Original image	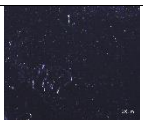	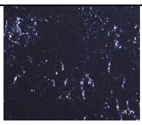	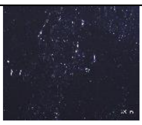
	Binary image	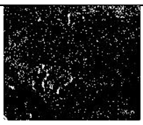	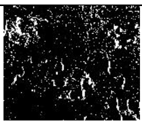	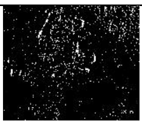
	Area Fraction (%)	5.5	6.8	5.8

Table S6: Images used to estimate the area fraction after ICI box pilling testing

Sample	Replicate	Image	Binary image	Area Fraction (%)
PES<br>§	1	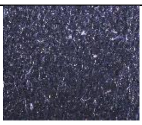	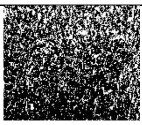	31.2
	2	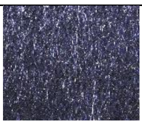	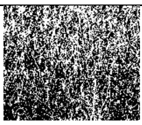	42.5
	3	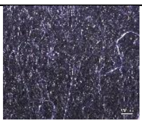	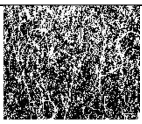	41.1
	4	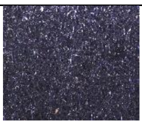	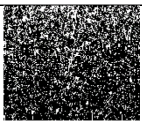	25.8

	5	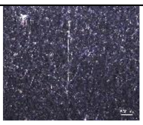	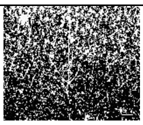	37.0
	6	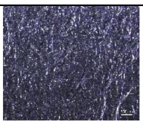	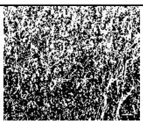	46.5
	7	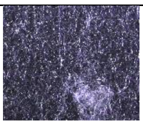	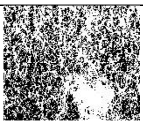	56.4
	8	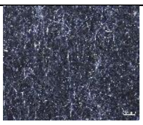	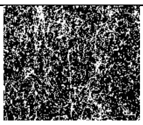	29.2
	9	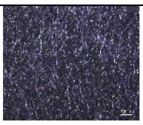	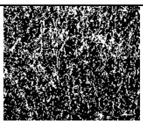	27.5
	10	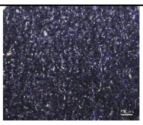	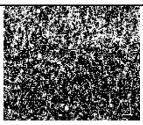	34.0
	11	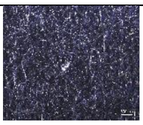	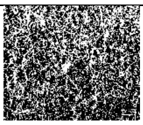	40.3
	12	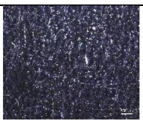	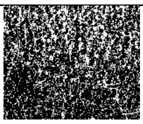	31.3
	13	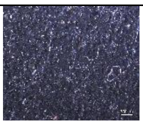	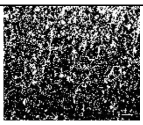	28.9
	14	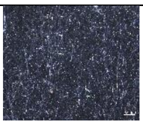	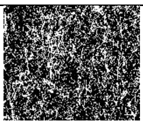	30.0
	15	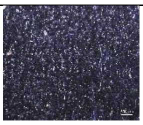	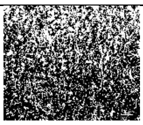	37.8
rPES-1	1	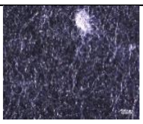	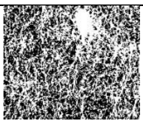	53.0
	2	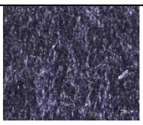	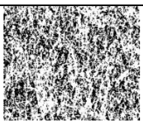	61.7

	3	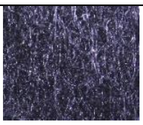	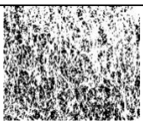	66.7
	4	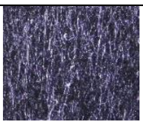	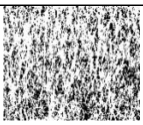	68.7
	5	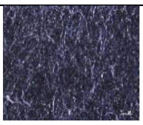	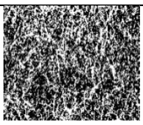	41.8
	6	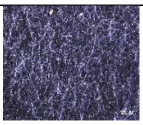	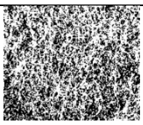	56.9
	7	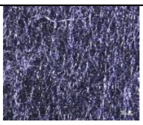	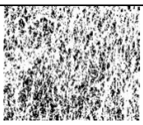	71.6
	8	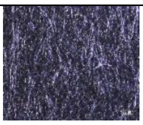	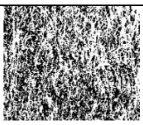	55.3
	9	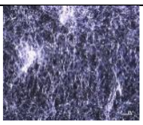	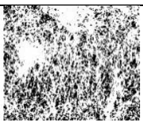	68.8
	10	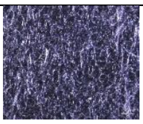	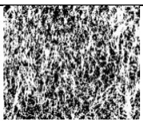	53.3
	11	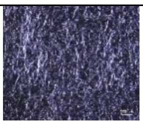	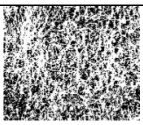	59.9
	12	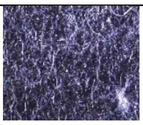	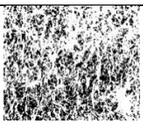	66.6
	13	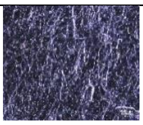	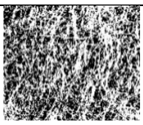	52.5
	14	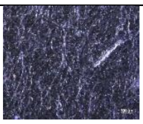	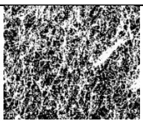	47.6
	15	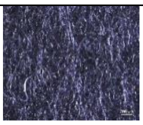	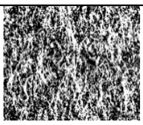	49.3

rPES-2	1	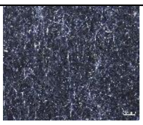	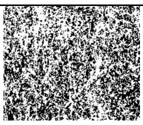	56.5
	2	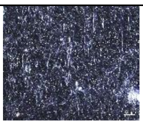	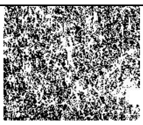	54.8
	3	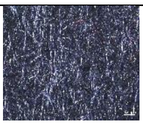	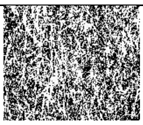	58.5
	4	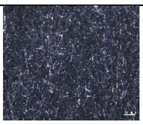	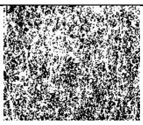	55.0
	5	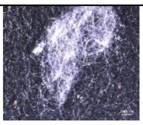	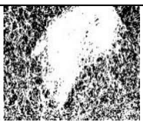	65.0
	6	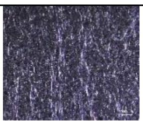	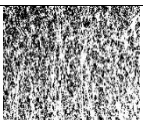	58.8
	7	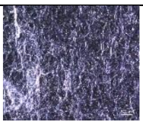	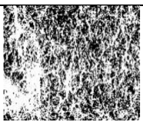	57.1
	8	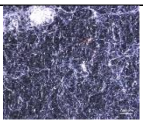	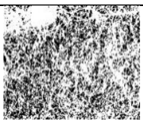	65.7
	9	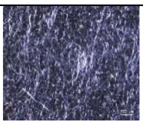	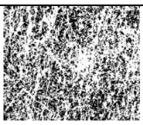	61.8
	10	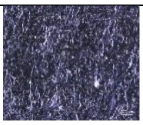	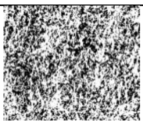	61.8
	11	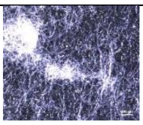	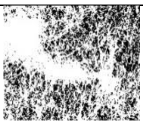	75.2
	12	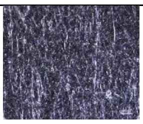	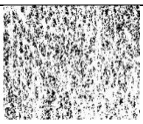	70.0
	13	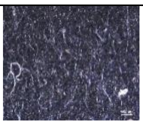	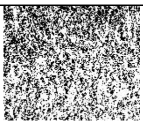	62.5

	14	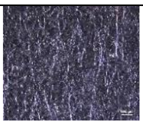	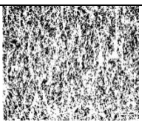	65.6
	15	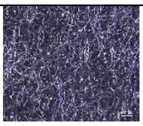	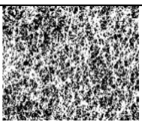	55.6
rPES-3	1	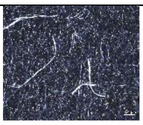	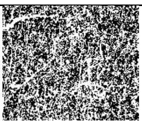	51.5
	2	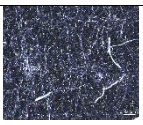	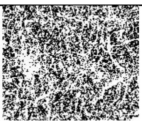	57.9
	3	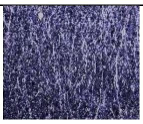	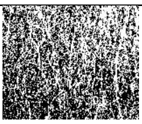	61.2
	4	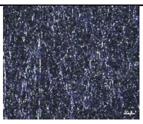	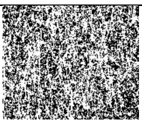	55.8
	5	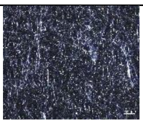	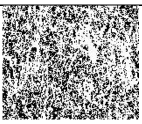	62.7
	6	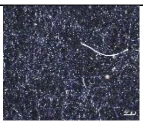	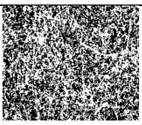	50.0
	7	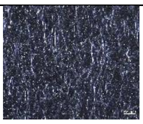	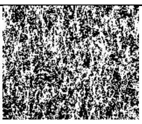	50.3
	8	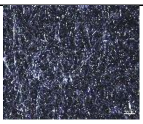	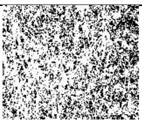	69.0
	9	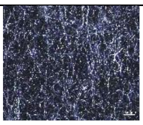	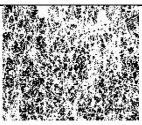	63.4
	10	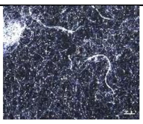	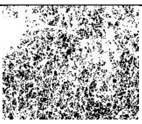	69.7
	11	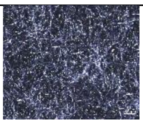	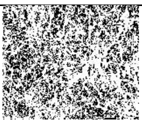	65.8

	12	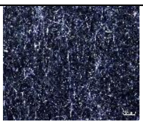	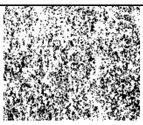	68.9
	13	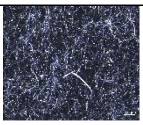	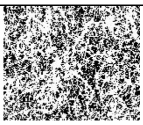	62.4
	14	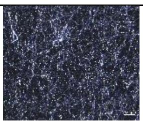	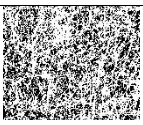	63.0
	15	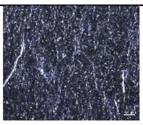	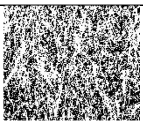	58.4

Table S7: Determination of fiber loss from fabric during washing

Sample	Replicate	Specimen Weight (g)	Filter + Petri glass combination weight before wash (g)	Filter + Petri glass combination weight after wash (g)	Mass of fiber release (g)	Fiber release (%)
PES	1	16.0312	40.1059	40.1062	0.0003	0.001871
	2	16.0553	39.5054	39.5056	0.0002	0.001246
	3	16.9593	39.5231	39.5234	0.0003	0.001769
	4	15.2269	39.6897	39.6900	0.0003	0.001970
rPES-1	1	11.0526	25.1585	25.1588	0.0003	0.002714
	2	12.7924	24.5166	24.5169	0.0003	0.002345
	3	10.3399	24.7493	24.7495	0.0002	0.001934
	4	11.8567	23.7100	23.7103	0.0003	0.002530
rPES-2	1	12.2022	39.5030	39.5039	0.0009	0.007376
	2	12.1936	41.5189	41.5199	0.0010	0.008201
	3	11.8338	40.8449	40.8459	0.0010	0.008450
	4	11.5149	39.5614	39.5620	0.0006	0.005211
rPES-3	1	13.8571	24.7503	24.7516	0.0010	0.007217
	2	15.5945	24.5152	24.5165	0.0013	0.008336
	3	11.9818	25.157	25.1585	0.0015	0.012519
	4	12.7813	23.711	23.7128	0.0018	0.014083

Table S8: Conversion of mean fiber loss from fabrics during washing

Sample	Fiber release (%)	g/kg	mg/kg
PES	0.001714 ± 0.000323	0.01714 ± 0.00323	17.14 ± 3.23
rPES	0.002381 ± 0.000334	0.02381 ± 0.00334	23.81 ± 3.34
rPES-2	0.007310 ± 0.001472	0.07310 ± 0.01472	73.10 ± 14.72
rPES-3	0.010539 ± 0.003285	0.10539 ± 0.03285	105.39 ± 32.85
